# A Metabolite Score of Unintentional Weight Loss Explained a Substantial Proportion of Associated Mortality and Mobility Limitation Risk in a Biracial Older Cohort

**DOI:** 10.1111/acel.70181

**Published:** 2025-08-06

**Authors:** Shanshan Yao, Megan M. Marron, Samaneh Farsijani, Iva Miljkovic, George C. Tseng, Ravi V. Shah, Venkatesh L. Murthy, Anne B. Newman

**Affiliations:** ^1^ University of Pittsburgh Pittsburgh Pennsylvania USA; ^2^ Vanderbilt University Medical Center Nashville Tennessee USA; ^3^ University of Michigan Ann Arbor Michigan USA

**Keywords:** metabolomics, mobility, mortality, unwanted weight loss

## Abstract

Unintentional weight loss (UWL) is related to mortality and mobility limitation. Here, we aimed to develop a metabolite‐based score for UWL and evaluate its prediction performance and explanation value for UWL‐related health outcomes. Participants from the Health, Aging and Body Composition (Health ABC) study with available metabolomics and valid follow‐ups were included (*N* = 2286). First, in the derivation group (*N* = 1200), 27 of the 77 metabolites associated with incident UWL (> 3% annual UWL vs. weight stable) were selected by LASSO‐logistic regression. The UWL metabolite score was calculated as a weighted sum of these 27 standardized metabolites, with higher scores indicating greater UWL risk. We then examined the standardized UWL metabolite score against all‐cause mortality and incident mobility limitation using Cox regression. Overall, older adults with a one‐SD higher UWL metabolite score had higher risks for mortality (1.44 [1.36, 1.52]) and mobility limitation (1.23 [1.15, 1.32]). The score also improved mortality prediction beyond traditional risk factors. Similar results were observed in the hold‐out test group (*n* = 1086). Furthermore, this score explained 28% of the UWL‐mortality relationship and 22% of the UWL‐mobility limitation relationship beyond lifestyle and medical history, respectively. The score also predicted higher mortality and mobility limitation among those with intentional weight loss and weight gain, demonstrating a good Out‐Of‐Distribution generalizability. This metabolomic characterization of UWL is predictive of key aging outcomes in the Health ABC participants and captures a substantial portion of the mortality and mobility limitation risks related to unintentional weight loss, further validating the importance of these metabolite signatures.

## Graphical abstract

A metabolite‐based score for short‐term unintentional weight loss was independently associated with mortality and mobility limitation risks in healthy older adults. It accounted for over 20% of the excess risk linked to unintentional weight loss, independent of lifestyle and medical history, suggesting a shared metabolic mechanism that remains unexplored.

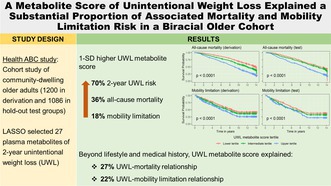

## 1 Introduction

Older adults often experience unintentional weight loss (Alibhai et al. 2005; Sahyoun et al. 2004), which is associated with a muscle mass decline of approximately 2% annually (Yao et al. 2025a)—double the rate of around 1% that was observed with “normal” aging (Goodpaster et al. 2006). As a key component of frailty, unintentional weight loss increases vulnerability to health conditions, mobility limitations, and mortality (Alibhai et al. 2005; De Stefani et al. 2018; Fried et al. 2001; Gregg et al. 2003; Ritchie et al. 2008; Sahyoun et al. 2004; Wannamethee et al. 2005). Our previous studies have indicated that metabolic changes were linked to subsequent unintentional weight loss (Yao et al. 2025b). This suggests that metabolic biomarkers could help better characterize and early identify older adults at risk for unintentional weight loss and related health outcomes. This knowledge may also guide the development of novel interventions to prevent unintentional weight loss and its related adverse outcomes in the older population.

Our prior study identified 77 metabolites, mostly lipids and lipid‐like molecules (e.g., TGs and phospholipids), organic acids (e.g., amino acids) and derivatives, and carbohydrates (e.g., hexose) and carbohydrate conjugates that potentially relate to subsequent unintentional weight loss during aging (Yao et al. 2025b). These metabolites suggest that altered amino acid and lipid metabolism pathways involving inflammation (Bozelli Jr. et al. 2021; Gonzalez‐Covarrubias 2013; Wallner et al. 2018), glucose metabolism or insulin resistance (Guasch‐Ferre et al. 2016; Long et al. 2020; Rhee et al. 2011), and kidney function (Drechsler et al. 2013; Tomaschitz et al. 2014; Yamaguchi et al. 2021) may precede unintentional weight loss in older adults. However, the extent to which these metabolites complement insights from demographics, lifestyles, health conditions, and widely used clinical biomarkers (e.g., cholesterol, fasting glucose, and cystatin C, etc.) remains unclear.

Here, we aimed to develop a novel metabolite score that is predictive of unintentional weight loss based on previously identified metabolites, with two primary objectives: (1) to assess its ability to predict short‐term unintentional weight loss and its related health outcomes; (2) to examine its ability to explain the heightened health risks observed following unintentional weight loss; and (3) to evaluate whether it provides additional explanatory value beyond single metabolites and commonly measured risk factors—including demographics, lifestyle, health conditions, and clinical biomarkers—in the Health, Aging and Body Composition (Health ABC) study. This approach ensures a clear temporality that the identified metabolites were measured before the onset of unintentional weight loss and subsequent health risks.

## 2 Methods

### 2.1 Population

The Health ABC cohort included 3075 Black and White older men and women aged 70–79 years enrolled between March 1997 and July 1998 from Pittsburgh, PA and Memphis, TN (Visser et al. 2005). At baseline, participants had to report being free of difficulty walking one‐quarter of a mile or climbing 10 steps, and with basic activities of daily living. In addition, they had no reported use of a walking aid at recruitment, no history of cancer treatment in the past 3 years, and no plans to move from the study area within 3 years after recruitment. Health ABC participants were followed up through annual in‐person examinations and semi‐annual telephone interviews. The institutional review boards of the University of Pittsburgh and the University of Tennessee approved the study. All participants provided written informed consent.

**FIGURE 1:**
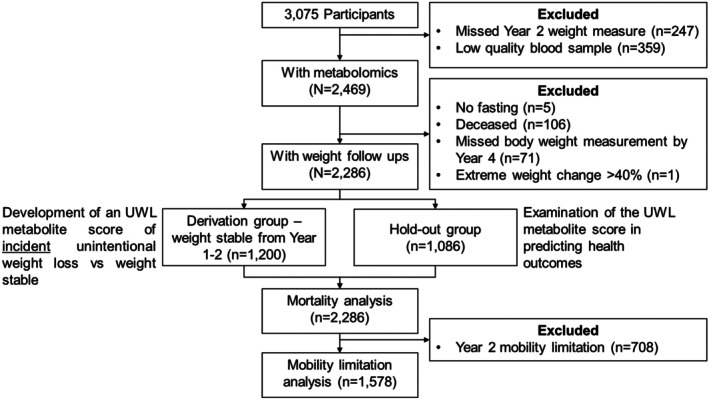
Study flowchart.

Of the 3075 participants recruited at baseline (i.e., Year 1, 1997/1998), 2828 returned for the Year 2 (1998/1999) clinic visit where blood samples and health conditions and lifestyles including body weight and dietary data were collected (Figure 1) Among them, high‐quality venipuncture samples were obtained from 2469 of these participants, making them eligible for metabolomic profiling. Participants with available metabolomics data were less likely to be Black, women, or smokers and had a lower prevalence of diabetes and depression compared to those without metabolomics data (Appendix Table A1). We then excluded five participants due to lack of overnight fasting prior to the visit to ensure data quality. Additionally, 178 participants were excluded either because they expired (*n* = 106) or had no follow‐up body weight measurement (*n* = 71) between Year 2 (1998/1999) and Year 4 (2000/2001), or exhibited extreme weight change exceeding 40% (10 times the standard deviation from the mean, *n* = 1). Thus, the final analysis included 2286 participants, among which 1200 were in the derivation group while 1086 were in the hold‐out (test) group. Participants included in this study were younger, less likely to be Black, men, smokers, or obese, and had more favorable health profiles compared to those who were excluded (Appendix Table A2).

### 2.2 Metabolomics

Metabolites were measured in plasma collected at the Year 2 visit after an overnight fast of ≥ 8 h using liquid chromatography‐mass spectrometry (LC–MS) methods at the Broad Institute (Cambridge, MA). Blood samples were stored at −80°C until profiling. Metabolite profiling methods and quality control have been published elsewhere (Yao et al. 2024; Yao et al. 2025c) and are reiterated in Appendix Methods. Missing values for metabolites were assumed to be below detectable limits and were imputed as 50% of the lowest value detected for the respective metabolite (Yao et al. 2024). Metabolite values were log‐transformed and standardized for further analyses.

### 2.3 UWL Metabolite Composite Score

In our previous study (Yao et al. 2025b), we identified 77 metabolites associated with the risk of incident 2‐year unintentional weight loss in previously weight stable Health ABC older adults. Among 1200 participants included in this previous report (Yao et al. 2025b) (derivation group including 220 participants experienced incident unintentional weight loss and 980 remained weight stable), we developed a composite metabolite score of unintentional weight loss, referred to as UWL metabolite score hereafter. To avoid overweighting highly correlated metabolites in the metabolite composite score, we used a LASSO‐logistic regression to select a subset from the 77 metabolites that were independently related to incident unintentional weight loss with no covariate adjustment. We then calculated the metabolite score in all 2286 participants as the sum of weighted and standardized metabolites. The metabolites were weighted by their standardized beta coefficients with unintentional weight loss from the LASSO‐logistic model. The UWL metabolites score was then standardized to mean 0, standard deviation 1 for following analyses. A higher UWL metabolite score indicates a higher potential risk for unintentional weight loss.

Based on the taxonomy of the metabolites in the Human Metabolome Database (HMDB), we also separated the UWL metabolite score into four subscores to determine if subsets of metabolites based on taxonomy group were accounted for in the results that we observed with the UWL metabolite score. We developed a UWL lipid score, a UWL organic acid score, a UWL sugar score, and a UWL “other” score, which included metabolites that were not in the classes of lipids and lipid‐like molecules, carbohydrates and carbohydrate conjugates, nor organic acids and derivatives.

### 2.4 Weight Change Groups

The weight change groups were determined using the same methods described in the previous study (Yao et al. 2025b). Briefly, participants were classified into weight stable (≤ 3% annual change), weight gain (> 3% annual increase), intentional weight loss (> 3% annual loss), and unintentional weight loss (> 3% annual loss) based on their intention to lose weight at Year 2 and the annual weight change from Year 2 to Year 4.

### 2.5 All‐Cause Mortality

Cause of death was adjudicated by a panel of clinicians who reviewed all hospital medical records, death certificates, proxy interviews, and autopsy reports (Kalogeropoulos et al. 2015). Date of death was taken from the death certificate or national death index. At the time of analysis, those alive were censored at their last interview date.

### 2.6 Incident Mobility Limitation

Persistent mobility limitation, “mobility limitation” hereafter, was defined as 2 consecutive reports of having difficulty walking one‐quarter mile or climbing 10 steps. Self‐reported physical function was assessed every 6 months. Reports must have involved the same function, that is, 2 reports of difficulty walking one‐quarter mile or 2 reports of difficulty climbing stairs. If participants missed a study visit, target dates for when the visit should have been completed were used to calculate the time to event or censorship. A final determination of limitation status was made from an interview or, if needed, a proxy interview and hospital records.

### 2.7 Covariates

Table 1 describes potential confounders including important risk factors of aging and aging related health outcomes such as demographics, health‐related lifestyles, and health conditions by weight loss status. All risk factors were collected at Year 1 or Year 2—the time of metabolomics, based on data availability, to facilitate a temporality requirement for confounding. Age, sex, race, highest level of education, and smoking behavior were self‐reported collected at Year 1. History or the presence of cardiovascular disease, hypertension, diabetes, and cancer were determined based on participants' self‐report of a physician diagnosis at Year 2. Participants were also categorized as having hypertension and diabetes if they were taking medication for these conditions. Peripheral artery disease, osteoporosis, depression, and pulmonary disease were collected at Year 1 only. The number of prescription medications used was collected through a medication inventory at Year 2. Body mass index (BMI; kg/m^2^) was calculated at Year 2 and categorized into < 25 kg/m^2^, 25–30 kg/m^2^, and ≥ 30 kg/m^2^. Total body fat was measured by dual‐energy X‐ray absorptiometry at Year 1. Mid‐thigh cross‐sectional muscle area was measured by Computed Tomography at Year 1. Self‐reported appetite or desire to eat during the past month was collected and was categorized into “Very good”, “Good”, and “Moderate to poor or fluctuated”. Daily calories (Kcal/d), protein (g/d), fat (g/d), and carbohydrates (g/d) intake from food at Year 2 were determined using a 108‐item interviewer administered food frequency questionnaire estimating usual nutrient intake over the past year (Marron et al. 2019). Daily fat (g/d) and protein intake (g/d) adjusted for total energy intake were calculated using the residual method (Willett et al. 1997). A Healthy Eating Index (HEI) (Lee et al. 2004) and physical activity energy expenditure (kcal/kg/week) (Yao et al. 2024) were also calculated. Physical activity was defined as energy expenditure in walking and climbing stairs at Year 2. To calculate energy expenditure in climbing stairs, we assigned 4.0 kcal/kg/h of stair climbing plus an additional 1.0 kcal/kg/h carrying a load like laundry, groceries, or an infant.

**TABLE 1:** Characteristics of participants from the health, aging and body composition study by weight change groups.

Characteristic	Overall *N* = 2286^a^	Unintentional weight loss *N* = 337^a^	Intentional weight loss *N* = 161^a^	Weight gain *N* = 429^a^	Weight stable *N* = 1359^a^	*p* ^b^
UWL metabolite score	0.0 (1.0)	0.5 (1.1)	0.0 (0.9)	0.0 (1.0)	−0.1 (1.0)	**< 0.001**
Age, years	74.6 (2.9)	74.6 (2.8)	74.6 (2.9)	74.6 (2.8)	74.6 (2.9)	> 0.9
Race and sex						**0.001**
White men	762 (33%)	98 (29%)	52 (32%)	123 (29%)	489 (36%)	
White women	678 (30%)	86 (26%)	46 (29%)	135 (31%)	411 (30%)	
Black men	361 (16%)	66 (20%)	21 (13%)	65 (15%)	209 (15%)	
Black women	485 (21%)	87 (26%)	42 (26%)	106 (25%)	250 (18%)	
More than high school education	1749 (77%)	231 (69%)	131 (82%)	326 (76%)	1061 (78%)	**0.001**
Smoker	199 (8.7%)	49 (15%)	7 (4.3%)	41 (9.6%)	102 (7.5%)	**< 0.001**
BMI category						**< 0.001**
< 25 kg/m^2^	764 (33%)	147 (44%)	16 (9.9%)	165 (38%)	436 (32%)	
25–30 kg/m^2^	958 (42%)	122 (36%)	84 (52%)	176 (41%)	576 (42%)	
≥ 30 kg/m^2^	564 (25%)	68 (20%)	61 (38%)	88 (21%)	347 (26%)	
Total whole body Fat (kg)	26.8 (8.7)	25.2 (8.4)	30.8 (8.6)	26.6 (8.7)	26.8 (8.6)	**< 0.001**
Total mid‐thigh muscle area (cm‐sq)	223.3 (55.7)	216.7 (51.3)	228.8 (57.9)	217.8 (56.2)	226.0 (56.2)	**0.005**
Current appetite						**< 0.001**
Very good	945 (42%)	118 (36%)	69 (43%)	169 (40%)	589 (44%)	
Good	840 (37%)	107 (33%)	68 (42%)	161 (38%)	504 (37%)	
Moderate to poor	473 (21%)	103 (31%)	24 (15%)	94 (22%)	252 (19%)	
Healthy eating index, 0–100	69.7 (12.1)	68.4 (12.6)	69.9 (11.9)	69.8 (12.3)	70.0 (12.0)	0.2
Total energy intake (Kcal/d)	1753.7 [1372.8‐2240.6]	1836.5 [1490.6‐2411.8]	1650.1 [1296.3‐2110.8]	1729.7 [1352.0‐2266.5]	1751.3 [1355.0‐2215.9]	**0.002**
Protein intake (g/d)^c^	65.5 [58.4–74.8]	66.0 [58.8–75.1]	68.0 [60.1–74.8]	66.3 [58.7–75.4]	65.0 [58.0–74.1]	0.11
Fat intake (g/d)^c^	71.0 [61.3–80.3]	70.5 [59.6–80.7]	69.8 [60.1–77.5]	70.1 [61.3–79.8]	71.4 [61.6–80.8]	0.2
Carbohydrate intake (g/d)^c^	248.2 [225.7–272.1]	250.0 [225.3–276.5]	249.2 [228.5–272.2]	248.9 [223.9–269.9]	247.0 [226.0–271.1]	0.6
Energy expenditure (Kcal/kg/week)	3.0 [0.3–9.4]	2.3 [0.1–8.1]	3.5 [0.8–8.0]	2.1 [0.1–9.2]	3.2 [0.5–10.0]	**0.019**
Cardiovascular disease	615 (27%)	96 (28%)	36 (22%)	102 (24%)	381 (28%)	0.2
Hypertension	1216 (53%)	183 (54%)	101 (63%)	233 (55%)	699 (51%)	**0.045**
Diabetes	894 (39%)	129 (38%)	70 (43%)	170 (40%)	525 (39%)	0.7
Cancer	420 (18%)	59 (18%)	24 (15%)	82 (19%)	255 (19%)	0.6
Peripheral artery disease	103 (4.6%)	18 (5.5%)	1 (0.6%)	25 (6.0%)	59 (4.4%)	**0.044**
Osteoporosis	231 (10%)	41 (12%)	20 (12%)	41 (9.7%)	129 (9.7%)	0.4
Depression	215 (9.4%)	24 (7.2%)	15 (9.3%)	49 (11%)	127 (9.4%)	0.3
Pulmonary disease	254 (11%)	47 (14%)	11 (6.9%)	51 (12%)	145 (11%)	0.10
Total prescription medications	3.0 [1.0–5.0]	3.0 [1.0–4.0]	3.0 [1.5–5.0]	3.0 [1.0–5.0]	3.0 [1.0–4.0]	**0.003**

*Note*: Bold values indicate *p* < 0.05.

^a^ Mean (SD), median [Q1‐Q3], or frequency (%).

^b^ One‐way analysis of means (not assuming equal variances); Pearson's chi‐squared test; Kruskal–Wallis rank sum test.

^c^ Macronutrient intakes adjusted for energy intake.

Furthermore, to help understand the relevance of our UWL metabolite score and its subscores with traditional biomarkers, we included clinical biomarkers that were measured in fasting blood samples. Traditional biomarkers included plasma triglycerides, high‐density lipoprotein cholesterol (HDL‐C), low‐density lipoprotein cholesterol (LDL‐C), and serum creatinine and cystatin C measured at Year 1, as well as plasma total cholesterol, serum glucose, interleukin‐6 (IL‐6) and C‐reactive protein (CRP) measured at Year 2 (Cesari et al. 2003).

### 2.8 Statistical Analysis

Mean (SD), median [Q1, Q3], and frequency (percent) were used to describe differences in continuous and categorical measures, as appropriate, by weight change groups and UWL metabolite score tertiles. Differences were tested using analysis of variance for continuous measures and chi‐square tests for categorical measures. To better understand the clinical relevance of the UWL metabolite scores, we also assessed the Spearman correlation between the established UWL metabolite score and clinical biomarkers.

We first examined the associations of the unadjusted UWL metabolite score and the subscores with unintentional weight loss using multinomial logistic regression models: Model 1—age, race, sex, BMI category, and study site adjusted; Model 2—additionally adjusted for lifestyle and known health conditions; Model 3—additionally adjusted for renal function (cystatin C). We also tested the race, sex, and categorized BMI interactions with the UWL metabolite score for unintentional weight loss to investigate potential subgroup‐specific relationships. We evaluated the predictive power of the UWL score for unintentional weight loss using area under the receiver operating characteristic (ROC) curves (AUCs).

Next, to investigate the long‐term weight trajectory across different weight change groups and UWL metabolite scores, we examined their relationship with percent body weight changes beyond Year 4 (specifically Years 5, 6, 8, 10, and 11) using linear mixed effects models and further tested the time interaction to assess these longitudinal patterns. We also conducted sensitivity analysis in the hold‐out (test) group to assess the robustness of these associations.

We then evaluated the associations of the UWL metabolite scores and the individual metabolites with mortality and incident mobility limitation using Cox proportional hazard regression models. We justified the proportional hazards assumption for Cox models using Schoenfeld residuals. We adjusted for potential confounders of the UWL metabolite score and outcomes using a forward stepwise approach: Model 1—age, race, sex, and study site adjusted; Model 2—additionally adjusted for lifestyle and known health conditions; Model 3—additionally adjusted for renal function (cystatin C). We tested for race‐ and sex‐interaction with the UWL metabolite score and conducted subgroup analyses by race and sex groups. We further stratified participants by weight change groups to assess how the UWL metabolite score predicts outcomes in those with and without unintentional weight loss and understand the score's performance outside its derivation group (Out‐Of‐Distribution generalization). In addition, the C‐index was used to examine the predictive power of the UWL metabolite score compared to other risk factors. The statistical significance of the difference between models was examined using the bootstrap method with 1000 resampling. We also conducted a sensitivity analysis in the hold‐out (test) group to assess the robustness of these associations and the predictive performance.

Then, to understand the UWL metabolite score's ability to explain the association between unintentional weight loss and health outcomes, we examined the percent attenuation in the association effect sizes between the demographics‐adjusted model (Model 0) and models after additionally adjusting for: (1) a single metabolite (Model 1), (2) the UWL metabolite score and the subscores (Model 2), (3) more commonly measured risk factors as mentioned above (Model 3), (4) the UWL metabolite score plus other risk factors (Model 4), and (5) traditional clinical biomarkers (Model 5). To examine whether the UWL metabolite score efficiently captures the risk of different outcomes related to unintentional weight loss, we used a LASSO‐Cox method to select subsets of metabolite and construct the outcome models for mortality and mobility limitation, respectively, and compared the explanation values with the UWL metabolite score. The percent attenuation was calculated using the formula (β_0_−β_1_)/β_0_ × 100% where β_0_ represents the beta coefficient estimate from the parsimonious model while β_1_ is the corresponding estimate from the further adjusted models. As a sensitivity analysis, we reran survival analyses using the accelerated failure time model and found similar results (data not shown).

For all the analyses, the significance level was set at 0.05. To account for multiple comparisons for individual metabolite analyses, we used a Benjamini‐Hochberg correction with 5% false discovery rate (FDR) (Benjamini and Hochberg 1995). All statistical analyses were conducted using R software version 4.2.1 (R Foundation for Statistical Computing).

## 3 Results

### 3.1 Participant Characteristics by Weight Change Groups

Participants had a mean age of 74.6 years (SD 2.9) at Year 2, with 37% self‐identifying as Black and 51% as women. Those with unintentional weight loss were more likely to be Black, smokers, less educated, and reported poorer appetite but higher calorie intake and lower physical activity levels, compared to other weight change groups (Table 1).

### 3.2 UWL Metabolite Score and Subscores

Among the 77 metabolites that were associated with incident unintentional weight loss, we selected 27 metabolites using a 10‐fold cross‐validated LASSO‐logistic regression model. Among these 27 metabolites, 11 were lipids and lipid‐like molecules (i.e., 4 phospholipids, 3 triglycerides, 3 fatty acids, and 1 acyl‐carnitine), 5 were organic acids and derivatives, 5 were carbohydrates and carbohydrate conjugates, and 6 were other metabolites (3 organoheterocyclic compounds, 2 nucleosides, nucleotides, and analogues, and carboxyibuprofen) (Appendix Table A3).

**FIGURE 2:**
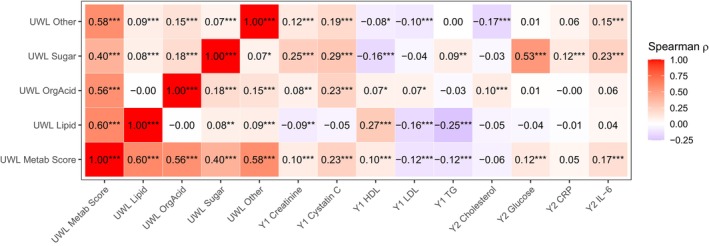
Spearman correlation between unintentional weight loss (UWL) metabolite score and subscores, and clinical biomarkers in the health, aging and body composition study participants.

The composite UWL metabolite score was significantly but moderately correlated with its subscores (Spearman Rs ranging from 0.40 to 0.60) and showed moderate correlations with Year 1 cystatin C (*R* = 0.23). In contrast, the subscores were only weakly correlated with each other (Rs < 0.2). The UWL lipid score was correlated with Year 1 HDL‐C (*R* = 0.27) and total triglycerides (*R* = 0.25); the UWL organic acid score was correlated with Year 1 cystatin C (*R* = 0.23); the UWL sugar score was correlated with Year 2 fasting glucose (*R* = 0.53), Year 1 cystatin C (*R* = 0.25), Year 1 creatinine (*R* = 0.29), and Year 2 IL‐6 (*R* = 0.23). The UWL “other” score was weakly correlated with clinical biomarkers (Rs ranging from −0.17 to 0.19). (Figure 2).

When comparing across tertiles of the UWL metabolite score, participants in the highest tertile (indicating the greatest potential risk for unintentional weight loss) were older and were more likely to be Black women. As expected, they were more likely to experience subsequent unintentional weight loss compared to the lowest tertile group (23% vs. 8.1%). They also had lower muscle mass and were more likely to report poorer appetite, lower dietary quality, and lower physical activity levels. Participants in the highest tertile also exhibited a higher prevalence of cardiovascular disease, hypertension, and diabetes and reported more prescription medication use than those in the lowest tertile (Table 2).

**TABLE 2:** Characteristics of participants from the health, aging and body composition study by UWL metabolite score tertiles.

Characteristic	Overall *N* = 2286^a^	UWL Metabolite Score			*p* ^b^
		Lower tertile *N* = 762^a^	Intermediate tertile *N* = 762^a^	Upper tertile *N* = 762^a^	
Weight change group					**< 0.001**
Weight stable	1359 (59%)	511 (67%)	466 (61%)	382 (50%)	
Unintentional weight loss	337 (15%)	62 (8.1%)	102 (13%)	173 (23%)	
Intentional weight loss	161 (7.0%)	56 (7.3%)	52 (6.8%)	53 (7.0%)	
Weight gain	429 (19%)	133 (17%)	142 (19%)	154 (20%)	
Age, years	74.6 (2.9)	74.3 (2.8)	74.5 (2.8)	75.1 (2.9)	**< 0.001**
Race and sex					0.057
White men	762 (33%)	282 (37%)	244 (32%)	236 (31%)	
White women	678 (30%)	227 (30%)	235 (31%)	216 (28%)	
Black men	361 (16%)	114 (15%)	116 (15%)	131 (17%)	
Black women	485 (21%)	139 (18%)	167 (22%)	179 (23%)	
More than high school education	1749 (77%)	636 (84%)	580 (76%)	533 (70%)	**< 0.001**
Smoker	199 (8.7%)	48 (6.3%)	64 (8.4%)	87 (11%)	**0.002**
BMI category					**0.003**
< 25 kg/m^2^	764 (33%)	244 (32%)	240 (31%)	280 (37%)	
25–30 kg/m^2^	958 (42%)	355 (47%)	315 (41%)	288 (38%)	
≥ 30 kg/m^2^	564 (25%)	163 (21%)	207 (27%)	194 (25%)	
Total whole body fat (kg)	26.8 (8.7)	26.3 (8.1)	27.4 (8.7)	26.8 (9.2)	**0.023**
Total mid‐thigh muscle area (cm‐sq)	223.3 (55.7)	230.0 (58.5)	221.7 (53.6)	218.1 (54.4)	**< 0.001**
Current appetite					**< 0.001**
Very good	945 (42%)	362 (48%)	317 (42%)	266 (36%)	
Good	840 (37%)	283 (37%)	292 (39%)	265 (36%)	
Moderate to poor	473 (21%)	113 (15%)	146 (19%)	214 (29%)	
Healthy eating index, 0–100	69.7 (12.1)	72.1 (11.5)	69.1 (12.0)	67.9 (12.5)	**< 0.001**
Total energy intake (Kcal/d)	1753.7 [1372.8‐2240.6]	1729.2 [1372.3‐2158.3]	1763.0 [1376.9‐2240.6]	1759.9 [1357.6‐2345.8]	0.3
Protein intake (g/d)^c^	65.5 [58.4–74.8]	65.2 [58.1–74.9]	65.7 [58.2–75.1]	65.7 [58.7–74.6]	> 0.9
Fat intake (g/d)^c^	71.0 [61.3–80.3]	71.6 [62.6–80.8]	70.1 [60.0–79.6]	70.8 [60.8–80.5]	**0.045**
Carbohydrate intake (g/d)^c^	248.2 [225.7–272.1]	245.7 [222.7–269.1]	251.5 [228.1–274.7]	246.1 [226.7–273.7]	**0.015**
Energy expenditure (Kcal/kg/week)	3.0 [0.3–9.4]	4.9 [1.0–11.7]	2.3 [0.2–8.6]	2.0 [0.1–7.8]	**< 0.001**
Cardiovascular disease	615 (27%)	176 (23%)	198 (26%)	241 (32%)	**< 0.001**
Hypertension	1216 (53%)	374 (49%)	413 (54%)	429 (56%)	**0.013**
Diabetes	894 (39%)	244 (32%)	311 (41%)	339 (44%)	**< 0.001**
Cancer	420 (18%)	151 (20%)	127 (17%)	142 (19%)	0.3
Peripheral artery disease	103 (4.6%)	30 (4.0%)	33 (4.4%)	40 (5.4%)	0.4
Osteoporosis	231 (10%)	82 (11%)	78 (10%)	71 (9.5%)	0.6
Depression	215 (9.4%)	76 (10%)	69 (9.1%)	70 (9.2%)	0.8
Pulmonary disease	254 (11%)	57 (7.5%)	91 (12%)	106 (14%)	**< 0.001**
Total prescription medications	3.0 [1.0–5.0]	2.0 [1.0–4.0]	3.0 [1.0–5.0]	3.0 [1.0–5.0]	**< 0.001**

*Note*: Bold values indicate *p* < 0.05.

^a^ Mean (SD) Median [Q1‐Q3], or Frequency (%).

^b^ One‐way analysis of means (not assuming equal variances); Pearson's chi‐squared test; Kruskal–Wallis rank sum test.

^c^ Macronutrient intakes adjusted for energy intake.

### 3.3 UWL Metabolite Score and Weight Change

As expected, older people with one‐SD higher UWL metabolite score had a 70% higher risk of experiencing unintentional weight loss, but not intentional weight loss nor weight gain, than being weight stable, independent of demographic, lifestyle, and health conditions. (Appendix Table A4) Subgroup analyses by race‐sex groups and BMI categories are shown in Appendix Tables A5 and A6, while the interactions were all not statistically significant. The UWL metabolite score also yielded better predictive power for unintentional weight loss when added to demographics and traditional risk factors (e.g., lifestyle and health conditions), although the improvement when adding to traditional risk factors was small in the hold‐out group. (Appendix Table A7).

**FIGURE A1:**
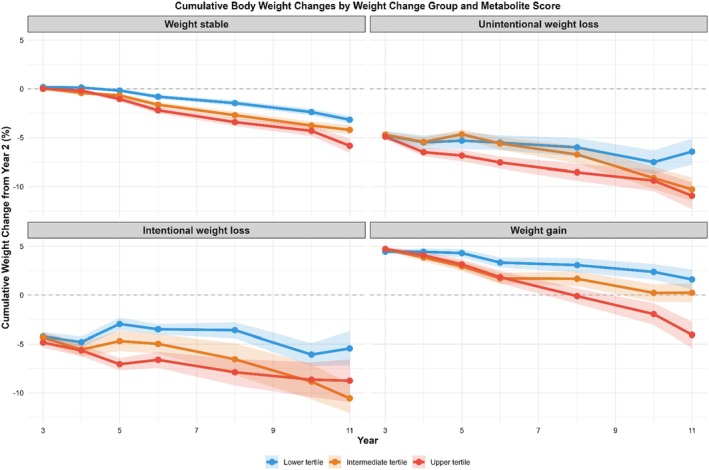
Body weight trajectory over time from Year 2 to Year 11 across weight change groups and the UWL metabolite score tertiles in the health, aging and body composition study participants.

In addition, the UWL metabolite score also stratified the long‐term weight loss tendency through Year 11, regardless of Year 2–4 weight change groups (Appendix Figure A1). Older adults with a higher UWL metabolite score lost more weight than those with a lower score for as long as 9 years after the metabolomics profiling. After adjusting for demographics, lifestyle, and health conditions, unintentional weight loss and higher UWL metabolite score remained significantly associated with greater weight loss beyond Year 4 through Year 11. The association between higher UWL metabolite score and weight loss was significantly stronger in later follow‐up years. Meanwhile, 2‐year weight gain was also associated with greater weight loss in later follow‐up years. Sensitivity analysis in the hold‐out group showed similar results (Appendix Table A8).

### 3.4 UWL Metabolite Score and Health Outcomes

**FIGURE A2:**
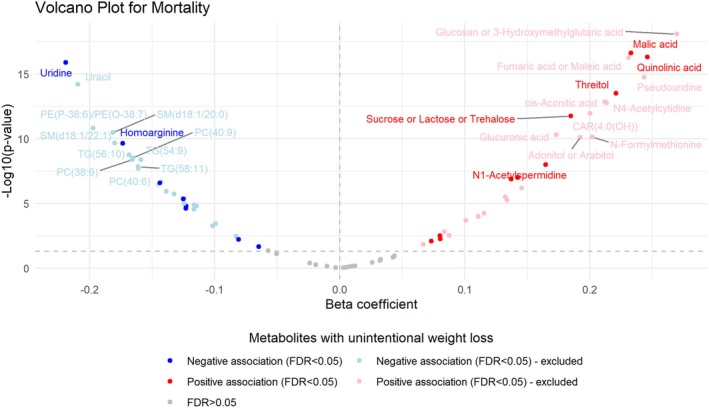
Association between unintentional weight loss associated metabolites with mortality in the health, aging and body composition study participants adjusting for age, race, sex, and study site.

**FIGURE A3:**
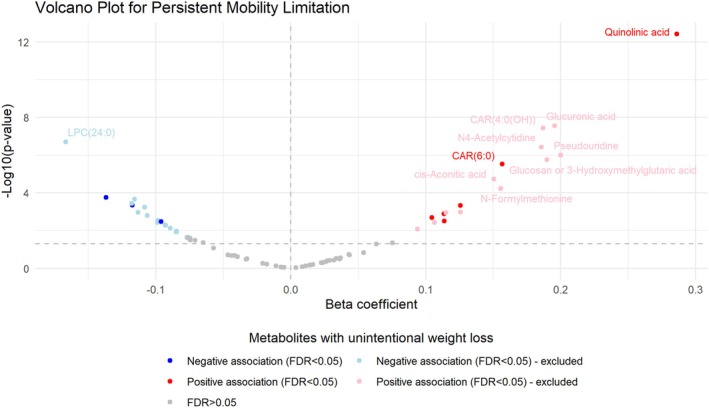
Association between unintentional weight loss associated metabolites with incident mobility limitation in the health, aging, and body composition study participants adjusting for age, race, sex, and study site.

As shown in Appendix Figures A2, A3, 61 and 36 metabolites out of the 77 metabolites (e.g., pseudouridine and quinolinic acid) were consistently associated with mortality and incident mobility limitation (FDRs < 0.05) after adjusting for age, race, sex, and study site, respectively. These metabolite associations were all in directions consistent with their associations with unintentional weight loss.

#### 3.4.1 All‐Cause Mortality

Among the 2286 participants, 1386 died during the follow‐up period, with a median follow‐up time of 12.3 years. Older participants experienced unintentional weight loss (hazard ratio (HR) = 1.52 [95% CI: 1.30, 1.77]), intentional weight loss (1.48 [1.20, 1.83]), and weight gain (1.22 [1.06, 1.41]) all showed higher subsequent mortality risk independent of demographics, lifestyles, and health conditions (Appendix Table A9).

Older adults with a 1‐SD higher UWL metabolite score had a 36% higher mortality (HR = 1.36 [1.28, 1.45]), regardless of their demographics, lifestyles, and health conditions. The UWL subscores were also related to higher mortality risk (HRs ranging from 1.14 to 1.21). Additionally adjusting for cystatin C attenuated 27% of the association for UWL sugar score and 22% for UWL organic acid score. Sensitivity analysis in the hold‐out group showed similar results with the associations slightly attenuated. (Table 3) Subgroup analyses are shown in Appendix Table A10 where the race‐ or sex‐interaction with the UWL metabolite score were not statistically significant.

**TABLE 3:** Association between unintentional weight loss (UWL) metabolite scores and health outcomes in the health, aging and body composition study participants.

Score	Model	Mortality		Mobility limitation	
		Overall *N* = 2286	Hold‐out group *N* = 1086	Overall *N* = 1578	Hold‐out group *N* = 516
UWL metabolite score	Model 1	**1.44 [1.36, 1.52]**	**1.39 [1.27, 1.53]**	**1.23 [1.15, 1.32]**	**1.22 [1.08, 1.38]**
	Model 2	**1.36 [1.28, 1.45]**	**1.31 [1.18, 1.45]**	**1.18 [1.09, 1.27]**	**1.18 [1.04, 1.36]**
	Model 3	**1.31 [1.23, 1.40]**	**1.25 [1.12, 1.39]**	**1.15 [1.06, 1.24]**	**1.17 [1.02, 1.34]**
UWL lipid score	Model 1	**1.15 [1.09, 1.22]**	1.10 [1.00, 1.20]	**1.07 [1.01, 1.15]**	1.02 [0.92, 1.14]
	Model 2	**1.14 [1.08, 1.21]**	1.08 [0.98, 1.18]	**1.08 [1.01, 1.16]**	1.05 [0.93, 1.18]
	Model 3	**1.14 [1.08, 1.21]**	1.08 [0.98, 1.19]	**1.08 [1.01, 1.16]**	1.05 [0.93, 1.18]
UWL organic acid score	Model 1	**1.26 [1.20, 1.33]**	**1.24 [1.13, 1.36]**	**1.17 [1.10, 1.26]**	1.21 [1.07, 1.36]
	Model 2	**1.21 [1.14, 1.28]**	**1.19 [1.08, 1.31]**	**1.15 [1.07, 1.24]**	1.16 [1.02, 1.31]
	Model 3	**1.16 [1.09, 1.23]**	**1.13 [1.02, 1.25]**	**1.13 [1.05, 1.22]**	1.14 [1.00, 1.30]
UWL sugar score	Model 1	**1.26 [1.19, 1.33]**	**1.26 [1.15, 1.38]**	**1.16 [1.08, 1.24]**	**1.23 [1.08, 1.39]**
	Model 2	**1.17 [1.10, 1.25]**	**1.15 [1.04, 1.28]**	1.06 [0.98, 1.15]	1.14 [0.98, 1.32]
	Model 3	**1.12 [1.04, 1.19]**	1.10 [0.98, 1.22]	1.03 [0.94, 1.11]	1.12 [0.96, 1.30]
UWL “other” score	Model 1	**1.25 [1.19, 1.32]**	**1.28 [1.17, 1.40]**	**1.10 [1.03, 1.17]**	1.11 [0.99, 1.24]
	Model 2	**1.20 [1.13, 1.26]**	**1.24 [1.12, 1.36]**	1.04 [0.97, 1.12]	1.06 [0.93, 1.21]
	Model 3	**1.16 [1.09, 1.23]**	**1.20 [1.08, 1.32]**	1.02 [0.95, 1.09]	1.05 [0.92, 1.20]

*Note:* Model 1: Adjusted for age, race, sex, study site, and BMI categories; Model 2: Model 1 + education, smoking, total fat mass, mid‐thigh muscle area, poor appetite, Healthy Eating Index, daily calorie intake, dietary protein and fat intake adjusting for energy intake, physical activity energy expenditure, prevalent/history of CVD, cancer, hypertension, diabetes, pulmonary disease, and number of medication use; Model 3: Model 2 + Cystatin C. Bold values indicate *p* < 0.05.

As shown in Appendix Table A11, adding the UWL metabolite score to the basal model resulted in a significantly higher predictive power for mortality (Model 2 vs. Model 0: C‐index = 0.645 vs. 0.607, *p* < 0.001), whereas adding the weight change groups improved the predictive power to a much smaller extent (Model 1 vs. Model 0: C‐index = 0.618 vs. 0.607, *p* < 0.001). In addition, additionally including the UWL metabolite score to traditional risk factors further improved the predictive power (Model 5 vs. Model 4: C‐index = 0.671 vs. 0.653, *p* < 0.001). The predictive power of the UWL metabolite score for mortality was similar when conducting the sensitivity analysis in the hold‐out group only.

In addition to the good predictive power, as shown in Table 4, the UWL metabolite score partially explained the association between unintentional weight loss and mortality. Further adjustment for the UWL metabolite score in addition to demographics (Model 0) attenuated the association by 39.3%, with the UWL lipid score attenuating the association by 7.1%, the UWL organic acid score by 16.3%, the UWL sugar score by 10.2%, and the UWL “other” score by 16.4%. Other commonly measured risk factors attenuated the association by 11.1%, and further adjustment for the UWL metabolite score led to an additional 27.6% attenuation. Clinical biomarkers attenuated the relationship by 15.7%. Individual metabolites attenuated the association by < 15% (mean 4.0%), and a subset of metabolites selected by LASSO‐Cox regression (*n* = 27) attenuated the association by 43.3% (Appendix Table A12).

**TABLE 4:** Attenuation of unintentional weight loss–outcome associations from Cox models after further adjusting for unintentional weight loss associated metabolites, metabolite score, and other risk factors in the health, aging and body composition study participants.

Model	Metabolites	Mortality (*N* = 2286)		Mobility limitation (*N* = 1578)	
		HR [95% CI]	% Attenuation	HR [95% CI]	% attenuation
Model 0	—	1.60 [1.38, 1.85]	Reference	1.25 [1.03, 1.51]	Reference
Model 1	CAR(6:0)	1.60 [1.38, 1.85]	0.5%	1.27 [1.05, 1.54]	−10.3%
Model 1	Lauric acid	1.59 [1.38, 1.84]	0.9%	1.25 [1.03, 1.51]	0.2%
Model 1	Juniperic acid	1.59 [1.37, 1.84]	1.6%	1.25 [1.03, 1.51]	−0.2%
Model 1	Sebacic acid	1.59 [1.38, 1.84]	1.0%	1.25 [1.03, 1.51]	0.0%
Model 1	LPC(22:5)	1.59 [1.38, 1.84]	1.0%	1.24 [1.03, 1.50]	1.1%
Model 1	LPE(20:4)	1.60 [1.39, 1.85]	−0.5%	1.25 [1.03, 1.51]	−0.1%
Model 1	Ornithine	1.60 [1.38, 1.85]	0.0%	1.25 [1.03, 1.52]	−2.0%
Model 1	Malic acid	1.53 [1.32, 1.77]	9.4%	1.25 [1.03, 1.51]	0.4%
Model 1	N1‐Acetylspermidine	1.58 [1.36, 1.82]	3.1%	1.24 [1.02, 1.50]	2.5%
Model 1	Sucrose or lactose or trehalose	1.56 [1.35, 1.80]	5.6%	1.23 [1.01, 1.48]	7.3%
Model 1	Sorbitol	1.57 [1.36, 1.81]	4.3%	1.24 [1.03, 1.50]	1.4%
Model 1	Threitol	1.56 [1.34, 1.80]	6.0%	1.25 [1.03, 1.51]	−1.2%
Model 1	Hexose	1.58 [1.37, 1.83]	2.0%	1.25 [1.03, 1.51]	−1.5%
Model 1	Quinolinic acid	1.56 [1.35, 1.81]	4.9%	1.23 [1.02, 1.49]	5.6%
Model 1	Carboxyibuprofen	1.59 [1.38, 1.84]	0.8%	1.24 [1.03, 1.50]	1.7%
Model 1	SM (d18:1/24:0)	1.56 [1.35, 1.80]	5.3%	1.22 [1.01, 1.48]	9.4%
Model 1	SM (d18:1/16:1)	1.57 [1.36, 1.81]	4.1%	1.24 [1.02, 1.50]	2.8%
Model 1	TG (53:3)	1.60 [1.38, 1.85]	0.4%	1.25 [1.03, 1.51]	0.1%
Model 1	TG (54:5)	1.60 [1.38, 1.85]	0.3%	1.25 [1.03, 1.51]	0.0%
Model 1	TG (56:8)	1.55 [1.34, 1.79]	6.8%	1.23 [1.01, 1.49]	7.0%
Model 1	Uridine	1.50 [1.29, 1.73]	14.1%	1.21 [1.00, 1.47]	11.8%
Model 1	ATP	1.60 [1.39, 1.85]	−0.3%	1.25 [1.03, 1.51]	−0.5%
Model 1	Tryptophan	1.57 [1.36, 1.82]	4.0%	1.24 [1.03, 1.50]	1.8%
Model 1	Homoarginine	1.56 [1.35, 1.81]	4.7%	1.24 [1.02, 1.50]	3.0%
Model 1	Glyceric acid	1.59 [1.38, 1.84]	0.8%	1.24 [1.03, 1.51]	0.9%
Model 1	1‐methyl nicotinamide	1.59 [1.37, 1.83]	1.9%	1.25 [1.03, 1.51]	0.3%
Model 1	Niacinamide	1.58 [1.37, 1.83]	2.1%	1.25 [1.03, 1.51]	−1.0%
Model 1 (mean)	—	—	4.0%	—	2.5%
Model 2	UWL metabolite score	1.33 [1.15, 1.54]	39.3%	1.17 [0.97, 1.42]	27.4%
Model 2_1	UWL lipid score	1.55 [1.34, 1.79]	7.1%	1.24 [1.02, 1.50]	3.6%
Model 2_2	UWL organic acid score	1.48 [1.28, 1.72]	16.3%	1.22 [1.01, 1.47]	10.4%
Model 2_3	UWL sugar score	1.53 [1.32, 1.76]	10.2%	1.23 [1.01, 1.48]	7.4%
Model 2_4	UWL “other” score	1.48 [1.28, 1.72]	16.4%	1.21 [1.00, 1.47]	12.3%
Model 3	Risk factors	1.52 [1.30, 1.77]	11.1%	1.17 [0.96, 1.43]	27.7%
Model 4	UWL metabolite score and risk factors	1.33 [1.14, 1.56]	38.7%	1.12 [0.91, 1.37]	50.2%
Model 5	Clinical biomarkers	1.49 [1.28, 1.73]	15.7%	1.19 [0.98, 1.45]	19.6%

*Note:* Model 0: adjusted for age, race, sex, BMI categories, and study site; Model 1: Model 0 + individual metabolites included in the UWL metabolite score; Model 2: Model 0 + UWL metabolite score; Model 3: Model 0 + risk factors including education, smoking, fat mass, muscle area, appetite, HEI, calorie intake, protein intake, fat intake, energy expenditure, CVD, cancer, hypertension, diabetes, pulmonary disease, number of medications; Model 4: Model 3 + risk factors; Model 5: Model 0 + Clinical biomarkers including total cholesterol, fasting glucose, CRP, IL‐6, cystatin C. Color shade indicates the magnitude of % attenuation.

#### 3.4.2 Incident Mobility Limitation

Among 1578 participants who were free of mobility limitation at Year 4 visit, 941 participants developed mobility limitation during the follow‐up period, with a median follow‐up time of 6.3 years. After adjusting for demographics, lifestyles, and health conditions, only weight gain (1.23 [1.03, 1.47]) was significantly associated with a higher risk for mobility limitation, while the associations for unintentional weight loss (HR = 1.17 [0.96, 1.43]) and intentional weight loss (1.07 [0.82, 1.40]) were not statistically significant (Appendix Table A9).

Older participants with higher UWL metabolite scores and the UWL organic acid subscores had greater hazards of mobility limitation independent of demographics, lifestyles, and health conditions. Sensitivity analysis in the hold‐out group showed similar results with the associations slightly attenuated. (Table 3) Subgroup analyses by race‐sex groups are shown in Appendix Table A10 while the race‐ or sex‐interaction with the UWL metabolite score was not statistically significant.

The mobility limitation predictive power for different combinations of predictors was compared in Appendix Table A13 using C‐index among the Health ABC participants. UWL metabolite score did not show higher prediction power than clinical biomarkers or traditionally measured risk factors for mobility limitation, although the model with traditional risk factors and the UWL metabolite score showed the best prediction power among all. The predictive power of the UWL metabolite score for mobility limitation was similar when conducting the sensitivity analysis in the hold‐out group only.

Further adjustment for the UWL metabolite composite score attenuated the association by 27.4%. Commonly measured risk factors attenuated the association by 27.7%, and further adjustment for the UWL metabolite score led to an additional 22.5% attenuation. Clinical biomarkers attenuated the association by 19.6%. (Table 4) Individual metabolites attenuated the association by less than 15% (mean 2.5%), and a subset of metabolites (*n* = 20) selected by LASSO‐Cox attenuated 26.7% of the association (Appendix Table A14).

### 3.5 Out‐Of‐Distribution Generalization: Stratification by Weight Change Groups

Stratified by weight change groups, we found that the relationships between the UWL metabolite score (and its subscores) and these outcomes in the weight gain and intentional weight loss groups were similar to those in the unintentional weight loss group. However, many associations were not statistically significant, likely due to the smaller effect size plus limited sample size. Specifically, among those with intentional weight loss, the risk of mortality was 28% higher (HR = 1.28 [0.98, 1.67]), and among those who gained weight, the risk was 29% higher (HR = 1.29 [1.12–1.49]). For mobility limitation, participants with higher scores in the weight gain group also had a higher risk (HR = 1.19 [0.97–1.46]) which was marginally significant (Appendix Table A15).

## 4 Discussion

Here, we developed a composite metabolite score of incident unintentional weight loss based on 27 metabolites that were individually related to unintentional weight loss in our previous work (Yao et al. 2025b). This UWL metabolite score was significantly and independently associated with greater long‐term weight loss, as well as higher risks of mortality and mobility limitation over more than 10 years. Furthermore, the UWL metabolite score explained 39.3% and 27.4% of the heightened mortality and mobility limitation risks associated with unintentional weight loss. Even after accounting for traditional risk factors including demographics, lifestyles, and health conditions, the UWL metabolite score added significant value (> 20%) in understanding risks related to unintentional weight loss.

Consistent with literature (De Stefani et al. 2018; Lee et al. 2005; Sahyoun et al. 2004; Wannamethee et al. 2005), we observed that older participants with unintentional weight loss exhibited a 60% higher risk of mortality and a 25% higher risk of mobility limitation regardless of age, race, and sex. Previously, our team has demonstrated a higher risk of mobility limitation over 2.5 years in Health ABC participants (Lee et al. 2005). In this work, we extended this report for a longer follow‐up (median 6.3 years). Other studies in older people reported 66%–115% higher mortality risk related to unintentional weight loss (De Stefani et al. 2018). The association with mortality remained strong and significant after adjusting for demographics, lifestyle risk factors, and health conditions in our study. Unintentional weight loss reflects aging and underlying health conditions, and the excess risk after adjusting for health status is likely due to undetected or undiagnosed disease (Wannamethee et al. 2005), warranting further investigation.

In this study, we developed a UWL metabolite score consisting of 27 metabolite correlates of 2‐year unintentional weight loss, which independently predicted long‐term weight loss, mortality, and mobility limitation. The UWL metabolite score also explained a notable portion of unintentional weight loss related risks of mortality and mobility limitation, which seems to be driven primarily by the organic acid and the “other” sub scores. In addition, we found that adjusting for the UWL metabolite score attenuated the association for mortality to a much larger extent than commonly measured risk factors, as well as clinical lipid‐, glucose‐, inflammation‐, and kidney function‐related biomarkers did. This suggests that the UWL metabolite score might be able to capture more nuanced metabolic changes shared by unintentional weight loss and subsequent health outcomes such as mortality. Furthermore, the UWL metabolite score significantly improved mortality prediction beyond demographics, lifestyles, and health conditions, highlighting its additional predictive value. In contrast, commonly measured risk factors appeared to attenuate the association between unintentional weight loss and mobility limitation to a similar extent as the UWL metabolite score did, suggesting a critical role of known health‐related behaviors and health conditions in the physical performance of older adults. Despite this, the UWL metabolite score continued to explain 23% of the remaining association beyond known risk factors, which again highlights its ability to capture metabolic risk beyond traditional known risk factors. We also observed that the combination of outcome‐based LASSO‐Cox selected metabolites did not provide substantially greater explanation value than the UWL metabolite score did for mortality and mobility limitation risks associated with unintentional weight loss. This suggests that the UWL metabolite score may have effectively captured metabolic risks underlying unintentional weight loss that are shared with mortality and mobility limitation.

Among the 27 metabolites included in our UWL metabolite score, 41% were classified as lipids and lipid‐like molecules (e.g., triglycerides and glycerophospholipids). The UWL lipid subscore showed a positive correlation with blood HDL‐C levels and a weak negative correlation with blood LDL‐C levels. This subscore was also consistently associated with higher subsequent risks of mortality and mobility limitation. These findings suggest that high blood HDL‐C levels, coupled with low LDL‐C levels, may contribute to a higher risk of unintentional weight loss and subsequent mortality. Our findings align with observational studies finding that older people with very high HDL‐C levels (over 80 mg/dL) had higher risks of death, cancer, dementia, and fractures (Hussain et al. 2023; Hussain, Robb, et al. 2024b; Hussain, Tonkin, et al. 2024; Ji and Duan 2024). High HDL‐C (“good” cholesterol) level may be indicative of impaired reverse cholesterol transport and antioxidant dysfunction, leading to adverse health outcomes in older adults (Hussain et al. 2023; Hussain, Robb, et al. 2024b; Ji and Duan 2024). In addition, previous studies also reported a J‐shaped relationship of all‐cause mortality with low LDL‐C (“bad” cholesterol) level (Akerblom et al. 2008; Wang et al. 2019). Low LDL‐C has been related to not only all‐cause mortality but also cancer, cancer mortality, and non‐CVD/non‐cancer mortality (Lu et al. 2024; Zhou et al. 2024). Therefore, a higher UWL lipid subscore in our study may have indicated a greater risk for non‐CVD health conditions such as cancer, which may lead to unintentional weight loss, mortality, and mobility limitation.

UWL subscore consisting of 5 organic acids and derivatives, among which 3 were amino acids (i.e., homoarginine, tryptophan, ornithine) was positively correlated with serum cystatin C level. This UWL organic acid subscore was also independently associated with higher odds of unintentional weight loss and explained a substantial portion of elevated risks for mortality and mobility limitation after weight loss. Additionally, adjusting for cystatin C substantially attenuated these associations. Together, these findings suggest an underlying mechanism of organic acid metabolism relating kidney function to unintentional weight loss and subsequent adverse outcomes. Indeed, these metabolites had been found to be associated with kidney function impairment in previous studies. The kidney was the major site of Arginine: Glycine Amidinotransferase Gene expression, which is related to the level of blood homoarginine (Cullen et al. 2006). Other studies also found that low homoarginine levels were closely associated with poorer kidney function as well as heart failure (Drechsler et al. 2013; Tomaschitz et al. 2014; Yamaguchi et al. 2021). Tryptophan metabolism was also associated with kidney disease, particularly through the kynurenine pathway. Evidence suggested that changes in metabolites of the tryptophan pathway were a consequence rather than a cause of poor kidney function (Cheng et al. 2020). Ornithine is related to the urea cycle, and its higher level has been related to chronic kidney disease and diabetic kidney (Thomson et al. 2001; Yamaguchi et al. 2021). Our findings aligned with previous reports that these metabolites are biomarkers for kidney dysfunction and, particularly, for related unintentional weight loss and subsequent health outcomes.

Moreover, we observed that the UWL sugar score consisting of 5 carbohydrates and carbohydrate conjugates (i.e., higher hexose, sorbitol, sucrose, threitol, and lower glyceric acid) explained the relationships of unintentional weight loss with mortality and, to a smaller extent, with mobility limitation. There was also a stronger positive correlation between the UWL sugar score and creatinine, cystatin C, as well as fasting glucose and IL‐6. This suggests that the UWL sugar subscore was related to kidney function, glucose metabolism, and inflammation, which is likely underlying unintentional weight loss and mortality. Sugar metabolites such as hexose and sucrose have been consistently related to impaired fasting glucose, prediabetes, and type 2 diabetes (Guasch‐Ferre et al. 2016). Studies have reported that weight loss was common after the diagnosis of type 2 diabetes (Looker et al. 2001); this might be due to disrupted glucose metabolism and energy metabolism. In addition, weight loss was more substantial among individuals not taking antidiabetic medication than those taking any medication, and those who took insulin showed the least weight loss (Looker et al. 2001). Our observation suggests that a greater risk for unintentional weight loss and subsequent adverse outcomes may be related to untreated or uncontrolled glucose dysregulation. Furthermore, within 5 years of the diabetes diagnosis, patients with nephropathy showed a greater decrease in body weight than those without nephropathy (Looker et al. 2001). Hyperglycemic states are known to be associated with kidney stress, oxidative stress, and inflammatory responses, elevating IL‐6 (de Rekeneire et al. 2006; Schrijvers et al. 2004). A higher UWL sugar score may signify greater metabolic strain on both kidney and glucose regulation pathways, promoting systemic inflammation, which was related to unintentional weight loss and mortality.

Among the metabolites included in the UWL “other” score, uridine exhibited a substantial impact on the associations of unintentional weight loss with mortality (14% attenuation) and mobility limitation (12% attenuation). Uridine is a pyrimidine nucleoside that plays an important role in several metabolic pathways such as cellular repair, mitochondrial energy production, and lipid and glucose metabolism. Abnormal levels of blood uridine have been related to diabetes and obesity (Yang et al. 2024), which again suggests a link of metabolic disorders with unintentional weight loss, mortality, and mobility limitation. Studies have also reported that uridine can rejuvenate aged human stem cells and promote regeneration of various tissues in vivo (Liu et al. 2022; Zeng et al. 2024). It may upregulate the FoxO signaling pathway and enhance self‐renewal while suppressing inflammation in aged hematopoietic stem cells (Zeng et al. 2024). Therefore, higher levels of blood uridine may also be related to lower risk of cancer and related health conditions (i.e., unintentional weight loss, mortality, and mobility limitation).

Unintentional weight loss is common in older adults, and it may signal dysregulated homeostasis and greater risks for mortality and mobility limitation. In the older population, lipid metabolism, glucose regulation, kidney dysfunction, and systemic inflammation appeared to be involved in the pathophysiology of unintentional weight loss. Metabolomics may have the potential to uncover metabolic pathways for therapeutic targets to prevent unintentional weight loss and its adverse outcomes. Maintaining healthy cholesterol levels, managing chronic kidney disease and diabetes to stabilize kidney function and blood glucose levels, as well as addressing systemic inflammation, may also help mitigate unintentional weight loss and its adverse health consequences. However, it is worth noting that the identified metabolites so far only partially explain the relationship between unintentional weight loss and adverse health outcomes, particularly mortality. More research is needed to determine the remaining elevated risk related to unintentional weight loss in older populations.

Interestingly, we found that the UWL metabolite score was also associated with higher risks of mortality and mobility limitation in those who experienced weight gain, intentional weight loss, and weight stability. This suggests that the UWL metabolite score may also reflect some shared metabolic risks related to mortality and mobility limitation that are not directly tied to unintentional weight loss. This also emphasizes the importance of the UWL metabolite score and its implicated pathways in healthy aging. It is worth noting that participants with weight gain or intentional weight loss were not included in the score development. Thus, our findings indicate that the UWL metabolite score has a good Out‐Of‐Distribution generalizability and could serve as a useful biomarker for identifying older adults at higher risk of adverse health outcomes, regardless of weight change patterns. Interventions targeting metabolic pathways represented in the UWL metabolite score may help mitigate risks of mortality and mobility limitation, even in individuals without unintentional weight loss. Additionally, we found that short‐term weight gain was significantly related to more long‐term weight loss in subsequent years. This suggests that the UWL metabolite score might be reflective of some subclinical metabolic changes due to aging or occult disease, which may lead to later weight loss and related health outcomes during aging.

A potential limitation of the current study was the lack of external replication for the association between the UWL metabolite score and mortality as well as mobility limitation in an independent sample of older adults. Future aging cohorts with metabolomics from different platforms (e.g., Metabolon) with similar or more comprehensive metabolomics profiling are encouraged to validate our findings. In addition, as a healthy older cohort, the Health ABC participants were at lower risk of many health conditions (e.g., incident cancer and dementia) compared to older adults of the same age. Therefore, we are at low power to examine the associations of unintentional weight loss, its related metabolites, and the UWL metabolite score with these important outcomes. Further exploration in aging cohorts with enough power is warranted. Despite this, there were several strengths of this study, including the well‐characterized cohort, internal validation for the UWL‐health outcome relationships, a large proportion of the Black older sample, clear temporality, and rigorous adjudication of mobility limitation.

## 5 Conclusion

In this study, we developed a composite score comprising 27 independent metabolites associated with incident unintentional weight loss over 2 years. This score emerged as a predictive tool for short‐term unintentional weight loss and a meaningful marker for long‐term weight loss over 9 years, as well as for unintentional weight loss‐related risks of mortality and mobility limitation. Indeed, the score also predicted adverse outcomes in older adults who did not have unintentional weight loss. This UWL metabolite score was a composite measure of metabolic status that captured complex information spanning different health domains related to lipid metabolism, glucose metabolism, kidney function, and inflammation. Nevertheless, a large proportion of the unintentional weight loss‐mortality association remains unexplained, and the causal role of selected metabolites is unknown. Future studies are warranted to provide a deeper characterization of unintentional weight loss and its shared mechanism with later adverse events, as well as to determine the timing of early diagnosis and intervention for unintentional weight loss and related conditions.

## Author Contributions

S.Y. conceptualized the analysis, analyzed data, constructed illustrations, and wrote the manuscript. M.M.M., S.F., I.M., G.C.T., R.V.S., and V.L.M. discussed, carefully reviewed, and revised the manuscript. A.B.N. supervised the study, conceptualized the study, reviewed, and revised the manuscript.

## Conflicts of Interest

In the past 12 months, Dr. Shah has served as a consultant for Amgen, Cytokinetics, and Thryv Therapeutics (with options ownership in Thryv). Dr. Shah is a co‐inventor on a patent for ex‐RNAs signatures of cardiac remodeling and a pending patent on proteomic signatures of fitness and lung and liver diseases. Dr. Murthy owns stock in General Electric, Cardinal Health, Pfizer, Amgen, Merck, Viatris, and Johnson & Johnson and stock options in Ionetix. He is a paid consultant for INVIA Medical Imaging Solutions & Siemens Healthineers. Dr. Murthy has received research support through his institution from Siemens Healthineers. Dr. Murthy is supported by the Melvyn Rubenfire Professorship in Preventive Cardiology. Drs. Murthy and Shah are also supported partly by grants from the National Institutes of Health and American Heart Association. Dr. Marron is supported by the National Institute on Aging K01‐AG‐075143. Dr. Newman is supported by the Pittsburgh Pepper Center P30‐AG024827. Dr. Farsijani is supported by a Career Development Award from the National Institute on Aging (K01 AG071855).

## Data Availability

Data included in this study is available at NIA (https://www.nia.nih.gov/healthabc‐study).

## Appendix A

### Metabolomic Profiling and Quality Control

Methods for metabolite profiling in the Health ABC study were identical to those in the Coronary Artery Risk Development in Young Adults (CARDIA) study at the Broad Institute (Cambridge, MA) which have been published previously (Abidi et al. 2020; Huang et al. 2016; Murthy et al. 2020; Yao et al. 2024). The Broad metabolomics platform enables robust, sensitive measures of a broad range of metabolites in human cohorts. Our methods use (1) state‐of‐the‐art high resolution/accurate mass (HRAM) mass spectrometry (MS) systems in full scan profiling mode to measure hundreds of known (and thousands of unknown metabolite) peaks or (2) sensitive triple quadrupole MS for targeted analyses of specific metabolites. We have confirmed identities of > 500 metabolites via authentic reference standards and identified > 1000 additional metabolites via mass matching to curated databases (METLIN89 and HMDB90). Because the metabolome consists of molecules with distinct properties (e.g., very polar organic acids to non‐polar lipids), we have developed/validated a series of procedures to provide a comprehensive metabolomic analysis of polar metabolites and lipids in < 100 μL of plasma.

Same as in CARDIA, we used four LC–MS platforms for metabolite detection: C8‐pos measures lipids of known identity and thousands of unknown peaks; C18‐neg measures free fatty acids, lipid‐derived mediators (eicosanoids), bile acids, and metabolites of intermediate polarity; HILIC‐pos measures amino acids, amino acid metabolites, acyl‐carnitines, dipeptides, and other cationic polar metabolites; HILIC‐neg measures sugars, organic acids, purines, pyrimidines, and other anionic polar metabolites. Metabolites in each sample were extracted from plasma using solutions containing internal standards, separated using liquid chromatography, and analyzed using high resolution, full scan mass spectrometry. Raw LC–MS data are acquired to a local computing resource and backed up on a Broad network resource (Isilon Systems). We performed processing of known metabolites using TraceFinder (v 3.2, Thermo Scientific) and confirmed compound identity using reference standards and reference samples included in each analysis queue. High resolution, non‐targeted data are processed using Progenesis QI software (Nonlinear Dynamics) to detect peaks, perform chromatographic retention time alignment, and integrate peak areas. Metabolites of confirmed identity are then annotated in the dataset and unknowns are “tagged” using their measured mass to charge ratio (m/z) and retention time. Quality control included the use of internal reference standards included in each sample (coefficient of variation [CV, standard deviation divided by mean] after log transformation of 0.2%), pooled reference plasma samples evaluated periodically throughout the run (median CV after log transformation of 0.3% across all 613 metabolites measured [IQR 0.2%–0.5%]), and evaluation of metabolite peaks to assure LC retention times and peak shapes. A sensitivity analysis has been done to account for batch effects due to lockdown during COVID‐19 while no evidence suggests a significant batch effect in metabolite measurement (Yao et al. 2024). Therefore, we analyzed the data without considering the batch effect.

Metabolite selection.

**TABLE A1:** Characteristics of participants from the health, aging and body composition study by metabolomics profiling availability. Characteristics from Year 2 visit are presented except where otherwise indicated.

Characteristic	Overall *N* = 2828	Metabolomics profiling		*p* ^a^
		No metabolomics *N* = 359	With metabolomics *N* = 2469	
	*N* (%), mean (SD), or median [Q1, Q3]			
Age, years	74.7 (2.9)	74.6 (2.8)	74.7 (2.9)	0.6
Race and sex				**< 0.001**
White men	894 (32%)	69 (19%)	825 (33%)	
White women	808 (29%)	98 (27%)	710 (29%)	
Black men	484 (17%)	73 (20%)	411 (17%)	
Black women	642 (23%)	119 (33%)	523 (21%)	
More than high school education	2143 (76%)	264 (74%)	1879 (76%)	0.3
Smoker	277 (9.8%)	50 (14%)	227 (9.2%)	**0.005**
BMI category				0.6
< 25 kg/m^2^	963 (34%)	120 (33%)	843 (34%)	
25–30 kg/m^2^	1192 (42%)	159 (44%)	1033 (42%)	
≥ 30 kg/m^2^	673 (24%)	80 (22%)	593 (24%)	
Total whole body fat (kg)	26.7 (8.7)	27.0 (8.5)	26.6 (8.7)	0.2
Total mid‐thigh muscle area (cm‐sq)	222.7 (55.5)	215.1 (54.2)	223.8 (55.6)	**0.006**
Appetite				0.2
Very good	1103 (41%)	103 (36%)	1000 (41%)	
Good	1025 (38%)	116 (40%)	909 (37%)	
Moderate to poor	595 (22%)	70 (24%)	525 (22%)	
Healthy eating index, 0–100	69.5 (12.1)	68.7 (12.1)	69.6 (12.1)	0.3
Calories (Kcal/d)	1758.9 [1364.5, 2266.6]	1765.3 [1299.5, 2326.9]	1758.9 [1375.0, 2251.0]	0.6
Daily protein intake adjusted for total calorie intake (g/d)	65.5 [58.4, 74.7]	64.9 [58.2, 73.2]	65.6 [58.4, 74.8]	0.3
Daily fat intake adjusted for total calorie intake (g/d)	71.1 [61.5, 80.5]	73.3 [64.0, 81.9]	70.8 [61.2, 80.3]	**0.019**
Daily carbohydrates intake adjusted for total calorie intake (g/d)	247.8 [226.0, 271.6]	245.4 [226.9, 266.6]	248.4 [225.9, 272.4]	0.14
Energy expenditure (Kcal/kg/Week)	2.8 [0.3, 9.2]	1.7 [0.2, 7.8]	3.0 [0.3, 9.4]	**0.006**
Cystatin C (mg/dL)	1.0 [0.9, 1.1]	1.0 [0.9, 1.2]	1.0 [0.9, 1.1]	0.3
Creatinine (mg/dL)	1.0 [0.9, 1.2]	1.0 [0.9, 1.1]	1.0 [0.9, 1.2]	0.5
Hemoglobin A1C (%)	6.1 [5.7, 6.6]	6.2 [5.8, 6.8]	6.1 [5.7, 6.6]	0.095
Total triglycerides (mg/dL)	118.0 [88.0, 165.0]	112.0 [82.5, 154.0]	119.0 [89.0, 166.0]	**0.009**
HDL (mg/dL)	51.0 [42.0, 63.0]	54.0 [43.0, 64.5]	51.0 [42.0, 63.0]	**0.020**
LDL (mg/dL)	120.0 [99.0, 143.0]	119.0 [95.0, 142.0]	120.0 [99.0, 143.0]	0.3
Total cholesterol (mg/dL)	204.0 [179.0, 229.0]	205.0 [174.0, 234.0]	204.0 [179.0, 228.0]	0.9
Fasting glucose (mg/dL)	94.0 [86.0, 106.0]	95.0 [85.0, 105.0]	94.0 [86.0, 106.0]	0.8
Interleukin‐6 (pg/mL)	2.4 [1.5, 4.0]	2.4 [1.6, 3.8]	2.4 [1.5, 4.1]	0.8
C‐reactive protein (μg/mL)	3.0 [1.3, 6.4]	3.8 [1.5, 7.4]	2.9 [1.2, 6.3]	**0.006**
Cardiovascular disease	800 (28%)	115 (32%)	685 (28%)	0.092
Hypertension	1529 (54%)	204 (57%)	1325 (54%)	0.3
Diabetes	1139 (40%)	173 (48%)	966 (39%)	**0.001**
Cancer	528 (19%)	68 (19%)	460 (19%)	0.9
Peripheral artery disease	145 (5.3%)	21 (6.1%)	124 (5.1%)	0.5
Osteoporosis	281 (10%)	39 (11%)	242 (10.0%)	0.5
Depression	277 (9.8%)	49 (14%)	228 (9.3%)	**0.008**
Pulmonary disease	324 (12%)	41 (12%)	283 (12%)	> 0.9
Total prescription medications	3.0 [1.0, 5.0]	3.0 [2.0, 5.0]	3.0 [1.0, 5.0]	**0.014**

^a^ One‐way analysis of means (not assuming equal variances); Pearson's chi‐squared test; Wilcoxon rank sum test. Bold values indicate *p* < 0.05.

**TABLE A2:** Characteristics of participants from the Health, Aging and Body Composition study by body weight follow‐ups. Characteristics from Year 2 visit are presented except for otherwise indicated.

Characteristic	Overall *N* = 2464	Weight follow‐up			*p* ^a^
		Deceased *N* = 106	Loss to follow‐up *N* = 72	With valid weight follow‐ups *N* = 2286	
	*N* (%), mean (SD), or mxedian [Q1, Q3]				
Age, years	74.7 (2.9)	75.4 (2.9)	74.7 (3.1)	74.6 (2.9)	**0.032**
Race and sex					**< 0.001**
White men	821 (33%)	40 (38%)	19 (26%)	762 (33%)	
White women	710 (29%)	20 (19%)	12 (17%)	678 (30%)	
Black men	411 (17%)	30 (28%)	20 (28%)	361 (16%)	
Black women	522 (21%)	16 (15%)	21 (29%)	485 (21%)	
More than high school education	1875 (76%)	76 (72%)	50 (69%)	1749 (77%)	0.2
Smoker	227 (9.2%)	18 (17%)	10 (14%)	199 (8.7%)	**0.005**
BMI category					**0.003**
< 25 kg/m^2^	840 (34%)	52 (49%)	24 (33%)	764 (33%)	
25–30 kg/m^2^	1032 (42%)	43 (41%)	31 (43%)	958 (42%)	
≥ 30 kg/m^2^	592 (24%)	11 (10%)	17 (24%)	564 (25%)	
Total whole body fat (kg)	26.7 (8.7)	23.7 (8.2)	26.1 (9.3)	26.8 (8.7)	**0.001**
Total Mid‐thigh muscle area (cm‐sq)	223.8 (55.5)	226.9 (52.9)	235.4 (52.1)	223.3 (55.7)	0.084
Appetite					**0.013**
Very good	1000 (41%)	27 (26%)	28 (40%)	945 (42%)	
Good	908 (37%)	46 (45%)	22 (31%)	840 (37%)	
Moderate to poor	523 (22%)	30 (29%)	20 (29%)	473 (21%)	
Healthy eating index, 0–100	69.6 (12.1)	68.7 (11.0)	66.3 (13.3)	69.7 (12.1)	0.085
Calories (Kcal/d)	1758.9 [1375.0‐2251.0]	1831.3 [1442.3‐2266.6]	1817.1 [1341.2‐2606.8]	1753.7 [1372.8‐2240.6]	0.5
Daily protein intake adjusted for total calorie intake (g/d)	65.6 [58.4–74.8]	66.9 [59.2–75.4]	66.6 [58.9–75.8]	65.5 [58.4–74.8]	0.7
Daily fat intake adjusted for total calorie intake (g/d)	70.8 [61.2–80.3]	69.5 [60.1–79.2]	69.7 [64.0–82.7]	71.0 [61.3–80.3]	0.6
Daily carbohydrates intake adjusted for total calorie intake (g/d)	248.4 [225.9–272.4]	251.7 [227.5–273.5]	242.8 [227.2–272.8]	248.2 [225.7–272.1]	0.6
Energy expenditure (Kcal/kg/Week)	3.0 [0.3–9.4]	3.3 [0.5–9.0]	1.6 [0.1–8.2]	3.0 [0.3–9.4]	0.7
Cystatin C (mg/dL)	1.0 [0.9–1.1]	1.1 [0.9–1.4]	1.0 [0.9–1.2]	1.0 [0.9–1.1]	**< 0.001**
Creatinine (mg/dL)	1.0 [0.9–1.2]	1.1 [1.0–1.3]	1.0 [0.9–1.3]	1.0 [0.9–1.2]	**< 0.001**
Hemoglobin A1C (%)	6.1 [5.7–6.6]	6.1 [5.8–6.8]	6.2 [5.7–6.6]	6.1 [5.7–6.6]	0.2
Total triglycerides (mg/dL)	119.0 [89.0–166.0]	122.0 [88.0–166.0]	108.0 [82.0–141.5]	119.0 [89.0–166.0]	0.2
HDL (mg/dL)	51.0 [42.0–63.0]	47.0 [38.0–59.0]	54.5 [44.5–64.5]	51.0 [42.0–63.0]	**0.039**
LDL (mg/dL)	120.0 [99.0–143.0]	116.5 [96.0–138.0]	119.5 [97.0–151.0]	120.0 [99.0–143.0]	0.3
Total cholesterol (mg/dL)	204.0 [179.0–228.0]	196.0 [168.0–228.0]	211.5 [182.0–228.0]	204.0 [180.0–228.0]	0.2
Fasting glucose (mg/dL)	94.0 [86.0–106.0]	93.0 [86.0–105.0]	95.5 [88.0–105.0]	94.0 [86.0–106.0]	0.8
Interleukin‐6 (pg/mL)	2.4 [1.5–4.1]	4.0 [2.2–7.6]	3.0 [2.0–4.0]	2.3 [1.5–3.9]	**< 0.001**
C‐reactive protein (μg/mL)	2.9 [1.2–6.3]	4.9 [1.9–11.3]	3.0 [1.1–6.9]	2.8 [1.2–6.2]	**< 0.001**
Cardiovascular disease	682 (28%)	41 (39%)	26 (36%)	615 (27%)	**0.008**
Hypertension	1321 (54%)	65 (61%)	40 (56%)	1216 (53%)	0.3
Diabetes	962 (39%)	41 (39%)	27 (38%)	894 (39%)	> 0.9
Cancer	459 (19%)	25 (24%)	14 (19%)	420 (18%)	0.4
Peripheral artery disease	122 (5.1%)	13 (13%)	6 (8.7%)	103 (4.6%)	**0.001**
Osteoporosis	241 (10.0%)	6 (5.9%)	4 (5.6%)	231 (10%)	0.2
Depression	228 (9.3%)	8 (7.6%)	5 (7.0%)	215 (9.4%)	0.7
Pulmonary disease	281 (11%)	19 (18%)	8 (11%)	254 (11%)	0.10
Total prescription medications	3.0 [1.0–5.0]	4.0 [2.0–6.0]	3.0 [1.0–5.0]	3.0 [1.0–5.0]	**0.007**

^a^ One‐way analysis of means (not assuming equal variances); Pearson's Chi‐squared test; Wilcoxon rank sum test. Bold values indicate *p* < 0.05.

**TABLE A3:** Weights for 27 metabolites associated with incident unintentional weight loss in 1200 previously weight stable Health ABC participants.

Metabolites	Platform	HMDB ID	Super class	Subclass	Weights
Ornithine	HILICp	HMDB0000214	Organic acids and derivatives	Amino acids, peptides, and analogues	0.149
Sebacic acid	C18n	HMDB0000792	Lipids and lipid‐like molecules	Fatty acids and conjugates	0.125
N1‐Acetylspermidine	HILICp	HMDB0001276	Organic acids and derivatives	Carboximidic acids	0.081
Carboxyibuprofen	C18n		Phenylpropanoids and polyketides	Not Available	0.072
Hexose	HILICn	HMDB0000122	Organic oxygen compounds	Carbohydrates and carbohydrate conjugates	0.072
Sucrose or Lactose or Trehalose	HILICn	HMDB0000258	Organic oxygen compounds	Carbohydrates and carbohydrate conjugates	0.071
LPC (22:5)	C8p	HMDB0010402	Lipids and lipid‐like molecules	Glycerophosphocholines	0.061
Malic acid	HILICn	HMDB0000156	Organic acids and derivatives	Beta hydroxy acids and derivatives	0.054
LPE (20:4)	C8p	HMDB0011517	Lipids and lipid‐like molecules	Glycerophosphoethanolamines	0.037
Quinolinic acid	HILICn	HMDB0000232	Organoheterocyclic compounds	Pyridinecarboxylic acids and derivatives	0.033
Threitol	HILICn	HMDB0004136	Organic oxygen compounds	Carbohydrates and carbohydrate conjugates	0.021
CAR (6:0)	HILICp	HMDB0000705	Lipids and lipid‐like molecules	Fatty acid esters	0.021
Lauric acid	C18n	HMDB0000638	Lipids and lipid‐like molecules	Fatty acids and conjugates	0.017
Sorbitol	HILICn	HMDB0000247	Organic oxygen compounds	Carbohydrates and carbohydrate conjugates	0.009
Juniperic acid	C18n	HMDB0006294	Lipids and lipid‐like molecules	Fatty acids and conjugates	0.002
1‐Methyl nicotinamide	HILICp	HMDB0000699	Organoheterocyclic compounds	Pyridinecarboxylic acids and derivatives	−0.019
SM (d18:1/16:1)	C8p	HMDB0240613	Lipids and lipid‐like molecules	Phosphosphingolipids	−0.026
SM (d18:1/24:0)	C8p	HMDB0011697	Lipids and lipid‐like molecules	Phosphosphingolipids	−0.046
ATP	HILICn	HMDB0000538	Nucleosides, nucleotides, and analogues	Purine ribonucleotides	−0.056
Homoarginine	HILICp	HMDB0000670	Organic acids and derivatives	Amino acids, peptides, and analogues	−0.059
Glyceric acid	HILICn	HMDB0000139	Organic oxygen compounds	Carbohydrates and carbohydrate conjugates	−0.060
Niacinamide	HILICp	HMDB0001406	Organoheterocyclic compounds	Pyridinecarboxylic acids and derivatives	−0.064
TG (56:8)	C8p	HMDB0005392	Lipids and lipid‐like molecules	Triradylcglycerols	−0.065
TG (54:5)	C8p	HMDB0005385	Lipids and lipid‐like molecules	Triradylcglycerols	−0.102
TG (53:3)	C8p	HMDB0043058	Lipids and lipid‐like molecules	Triradylcglycerols	−0.121
Uridine	HILICn	HMDB0000296	Nucleosides, nucleotides, and analogues	Not Available	−0.178
Tryptophan	HILICp	HMDB0000929	Organic acids and derivatives	Amino acids, peptides, and analogues	−0.208

*Note:* Metabolites were organized by taxonomy super class according to the Human Metabolome Database. Pink shade indicates a positive association between metabolites and unintentional weight loss while blue shade indicates a negative association.

**TABLE A4:** Association between unintentional weight loss (UWL) metabolite scores and weight change groups in the health, aging and body composition study.

Metabolite score	Model	Unintentional weight loss	Intentional weight loss	Weight gain
UWL metabolite score	Model 1	**1.78 [1.57, 2.01]**	1.13 [0.94, 1.36]	**1.19 [1.06, 1.34]**
	Model 2	**1.70 [1.48, 1.95]**	1.13 [0.94, 1.37]	**1.13 [1.00, 1.29]**
	Model 3	**1.66 [1.45, 1.91]**	1.11 [0.92, 1.34]	1.09 [0.96, 1.24]
UWL lipid subscore	Model 1	**1.28 [1.14, 1.45]**	0.89 [0.75, 1.06]	1.11 [0.99, 1.24]
	Model 2	**1.24 [1.09, 1.41]**	0.90 [0.75, 1.08]	1.07 [0.96, 1.21]
	Model 3	**1.24 [1.09, 1.41]**	0.90 [0.75, 1.08]	1.07 [0.95, 1.21]
UWL organic acid subscore	Model 1	**1.50 [1.33, 1.69]**	**1.21 [1.02, 1.43]**	**1.18 [1.05, 1.32]**
	Model 2	**1.46 [1.29, 1.67]**	1.18 [0.99, 1.41]	**1.15 [1.02, 1.29]**
	Model 3	**1.42 [1.25, 1.61]**	1.16 [0.98, 1.39]	1.12 [0.99, 1.26]
UWL sugar subscore	Model 1	**1.33 [1.18, 1.50]**	1.02 [0.86, 1.22]	1.01 [0.90, 1.14]
	Model 2	**1.38 [1.19, 1.60]**	1.01 [0.85, 1.21]	0.96 [0.85, 1.10]
	Model 3	**1.33 [1.15, 1.53]**	0.98 [0.82, 1.17]	0.91 [0.79, 1.03]
UWL “other” subscore	Model 1	**1.41 [1.25, 1.59]**	**1.20 [1.01, 1.43]**	1.06 [0.94, 1.19]
	Model 2	**1.32 [1.16, 1.50]**	**1.21 [1.02, 1.44]**	1.03 [0.91, 1.16]
	Model 3	**1.28 [1.12, 1.45]**	**1.20 [1.01, 1.44]**	1.00 [0.89, 1.13]

*Note:* Model 1: Adjusted for age, race, sex, study site, and BMI categories; Model 2: Model 1 + education, smoking, total fat mass, mid‐thigh muscle area, poor appetite, Healthy Eating Index, daily calorie intake, dietary protein and fat intake adjusting for energy intake, physical activity energy expenditure, prevalent/history of CVD, cancer, hypertension, diabetes, pulmonary disease, and number of medication use; Model 3: Model 2 + Cystatin C. Bold values indicate *p* < 0.05.

**TABLE A5:** Association between unintentional weight loss (UWL) metabolite scores and weight change groups in the health, aging and body composition study participants stratified by race and sex groups.

Strata	Metabolite score	Unintentional weight loss	Intentional weight loss	Weight gain
White men	UWL metabolite score	**1.77 [1.37, 2.27]**	1.16 [0.82, 1.64]	0.89 [0.69, 1.14]
	UWL lipid score	**1.31 [1.04, 1.63]**	0.90 [0.66, 1.24]	0.95 [0.77, 1.18]
	UWL organic acid score	**1.30 [1.01, 1.68]**	1.05 [0.75, 1.48]	0.91 [0.71, 1.17]
	UWL sugar score	**1.41 [1.07, 1.86]**	1.16 [0.83, 1.64]	0.91 [0.71, 1.17]
	UWL “other” score	**1.45 [1.15, 1.83]**	1.34 [0.98, 1.83]	0.97 [0.76, 1.22]
White women	UWL metabolite score	**1.95 [1.47, 2.59]**	1.02 [0.70, 1.48]	1.23 [0.98, 1.54]
	UWL lipid score	1.18 [0.92, 1.51]	0.93 [0.67, 1.30]	1.11 [0.90, 1.36]
	UWL organic acid score	**1.59 [1.23, 2.04]**	1.02 [0.73, 1.44]	1.22 [0.99, 1.50]
	UWL sugar score	**1.59 [1.17, 2.15]**	0.83 [0.56, 1.22]	1.00 [0.78, 1.28]
	UWL “other” score	**1.53 [1.17, 2.00]**	1.24 [0.86, 1.80]	1.06 [0.84, 1.33]
Black men	UWL metabolite score	**1.73 [1.24, 2.41]**	1.18 [0.75, 1.85]	1.34 [0.97, 1.85]
	UWL lipid score	1.06 [0.76, 1.46]	0.80 [0.51, 1.27]	1.14 [0.82, 1.59]
	UWL organic acid score	**1.81 [1.29, 2.56]**	1.61 [0.98, 2.67]	1.60 [1.15, 2.23]
	UWL sugar score	**1.62 [1.14, 2.29]**	1.30 [0.82, 2.07]	1.28 [0.91, 1.80]
	UWL “other” score	1.33 [0.98, 1.80]	1.08 [0.70, 1.66]	0.94 [0.69, 1.28]
Black women	UWL metabolite score	**1.62 [1.21, 2.18]**	1.17 [0.76, 1.80]	1.10 [0.84, 1.44]
	UWL lipid score	**1.46 [1.07, 2.00]**	0.92 [0.60, 1.41]	1.14 [0.86, 1.50]
	UWL organic acid score	**1.41 [1.09, 1.83]**	1.31 [0.93, 1.84]	1.10 [0.86, 1.40]
	UWL sugar score	1.19 [0.88, 1.62]	0.93 [0.65, 1.35]	0.88 [0.67, 1.16]
	UWL “other” score	1.10 [0.83, 1.47]	1.08 [0.74, 1.57]	1.00 [0.77, 1.28]

*Note:* Models adjusted for age, study site, Year 2 body weight, smoking, total fat mass, mid‐thigh muscle area, poor appetite, healthy eating index, daily calorie intake, dietary protein and fat intake adjusting for energy intake, physical activity energy expenditure, prevalent/history of CVD, cancer, hypertension, diabetes, pulmonary disease, and number of medication use. Bold values indicate *p* < 0.05.

**TABLE A6:** Association between unintentional weight loss (UWL) metabolite scores and weight change groups in the health, aging and body composition study participants stratified by BMI categories.

Strata	Metabolite score	Unintentional weight loss	Intentional weight loss	Weight gain
BMI: < 25 kg/m^2^	UWL metabolite score	**1.62 [1.30, 2.01]**	1.32 [0.77, 2.26]	**1.34 [1.09, 1.64]**
	UWL lipid score	1.14 [0.93, 1.40]	0.67 [0.38, 1.17]	**1.26 [1.04, 1.53]**
	UWL organic acid score	**1.49 [1.21, 1.84]**	**1.88 [1.17, 3.03]**	**1.23 [1.01, 1.50]**
	UWL sugar score	**1.29 [1.01, 1.64]**	0.81 [0.45, 1.45]	0.83 [0.66, 1.04]
	UWL “other” score	**1.35 [1.10, 1.64]**	1.35 [0.83, 2.20]	1.18 [0.98, 1.44]
BMI: 25–30 kg/m^2^	UWL metabolite score	**1.79 [1.42, 2.24]**	1.11 [0.85, 1.45]	0.98 [0.80, 1.19]
	UWL lipid score	**1.43 [1.16, 1.76]**	1.05 [0.82, 1.34]	0.99 [0.82, 1.19]
	UWL organic acid score	**1.37 [1.10, 1.70]**	1.04 [0.80, 1.34]	1.04 [0.86, 1.27]
	UWL sugar score	**1.46 [1.14, 1.88]**	1.07 [0.79, 1.43]	1.16 [0.93, 1.43]
	UWL “other” score	**1.33 [1.07, 1.65]**	1.08 [0.84, 1.39]	0.86 [0.71, 1.05]
BMI: ≥ 30 kg/m^2^	UWL metabolite score	**2.01 [1.46, 2.77]**	1.16 [0.83, 1.61]	1.06 [0.79, 1.42]
	UWL lipid score	1.26 [0.92, 1.71]	0.77 [0.56, 1.07]	0.92 [0.70, 1.21]
	UWL organic acid score	**1.65 [1.25, 2.16]**	1.20 [0.90, 1.59]	1.17 [0.91, 1.50]
	UWL sugar score	**1.49 [1.08, 2.06]**	1.03 [0.74, 1.42]	0.87 [0.66, 1.16]
	UWL “other” score	**1.38 [1.02, 1.87]**	**1.38 [1.03, 1.86]**	1.11 [0.84, 1.46]

*Note:* Models adjusted for age, race, sex, study site, smoking, total fat mass, mid‐thigh muscle area, poor appetite, Healthy Eating Index, daily calorie intake, dietary protein and fat intake adjusting for energy intake, physical activity energy expenditure, prevalent/history of CVD, cancer, hypertension, diabetes, pulmonary disease, and number of medication uses. Bold values indicate *p* < 0.05.

**TABLE A7:** Predictive power for unintentional weight loss using demographics, the UWL metabolite score, clinical biomarkers, and traditional risk factors using area under the receiver operating characteristics curve (AUC) among Health ABC participants.

Model	Predictors	AUC (95% CI)	
		Overall	Hold‐out group
Model 0	Age, race, sex, study site	0.568 (0.534–0.601)**	0.610 (0.556–0.663)**
Model 1	Age, race, sex, study site, traditional measured risk factors	0.652 (0.620–0.684)*	0.685 (0.636–0.734)*
Model 2	Age, race, sex, study site, clinical biomarkers	0.578 (0.544–0.612)**	0.618 (0.563–0.674)**
Model 3	Age, race, sex, study site, UWL metabolite score	0.659 (0.627–0.692)*	0.632 (0.578–0.687)**
Model 4	Age, race, sex, study site, traditional risk factors, UWL metabolite score	0.697 (0.667–0.728)*, **	0.694 (0.645–0.744)*

*Note:* Clinical biomarkers included total cholesterol, fasting glucose, CRP, IL‐6, cystatin C; Traditional risk factors included education, smoking, appetite, HEI, calorie intake, energy expenditure, CVD, cancer, hypertension, diabetes, pulmonary disease, number of medications.

Abbreviation: CI = confidence interval.

* *p* < 0.05 compared to Model 0.

** *p* < 0.05 compared to Model 1.

**TABLE A8:** Weight change groups (Year 2–4) and the UWL metabolite score in relation to long‐term percent weight change from Year 4–11 among Health ABC participants.

Effects	Overall		Hold‐out group	
	Main effect	Time interaction	Main effect	Time interaction
Models with either weight change or the UWL metabolite score				
Weight change groups (Year 2–4)				
Unintentional weight loss	**−4.90 [−5.54, −4.26]**	−0.21 [−0.43, 0.01]	**−5.01 [−6.25, −3.79]**	−0.03 [−0.44, 0.37]
Intentional weight loss	**−3.66 [−4.51, −2.82]**	0.04 [−0.23, 0.32]	**−4.09 [−5.63, −2.55]**	−0.02 [−0.49, 0.45]
Weight gain	**3.78 [3.22, 4.34]**	**−0.20 [−0.38, −0.02]**	**4.91 [3.89, 5.93]**	−0.06 [−0.38, 0.25]
UWL metabolite score (Year 2)				
UIWL metabolite score	**−0.90 [−1.16, −0.63]**	**−0.23 [−0.30, −0.15]**	**−1.13 [−1.66, −0.61]**	−0.14 [−0.28, 0.00]
Combined model with both weight change and the original UWL metabolite score				
Weight change groups				
Unintentional weight loss	**−4.63 [−5.27, −3.98]**	−0.10 [−0.32, 0.11]	**−4.91 [−6.13, −3.70]**	−0.02 [−0.42, 0.38]
Intentional weight loss	**−3.61 [−4.45, −2.77]**	0.06 [−0.21, 0.33]	**−4.02 [−5.55, −2.50]**	−0.00 [−0.47, 0.46]
Weight gain	**3.84 [3.28, 4.39]**	−0.18 [−0.36, 0.00]	**4.87 [3.87, 5.88]**	−0.06 [−0.38, 0.25]
UWL metabolite score				
UWL metabolite score	**−0.64 [−0.86, −0.41]**	**−0.22 [−0.30, −0.15]**	**−0.95 [−1.40, −0.50]**	**−0.14 [−0.28, −0.00]**

*Note:* Models adjusted for age, study site, Year 2 body weight, smoking, total fat mass, mid‐thigh muscle area, poor appetite, Healthy Eating Index, daily calorie intake, dietary protein and fat intake adjusting for energy intake, physical activity energy expenditure, prevalent/history of CVD, cancer, hypertension, diabetes, pulmonary disease, and number of medication use. Main effects were extracted from models without adjusting for follow‐up period. Bold values indicate *p* < 0.05.

**TABLE A9:** Weight change groups (Year 2–4) and subsequent health outcomes.

Outcome	Model	Unintentional weight loss	Intentional weight loss	Weight gain
Mortality	Model 1	**1.60 [1.38, 1.85]**	**1.40 [1.14, 1.72]**	**1.23 [1.07, 1.42]**
	Model 2	**1.52 [1.30, 1.77]**	**1.48 [1.20, 1.83]**	**1.22 [1.06, 1.41]**
	Model 3	**1.49 [1.28, 1.74]**	**1.44 [1.16, 1.78]**	**1.19 [1.03, 1.38]**
Mobility limitation	Model 1	**1.25 [1.03, 1.51]**	1.11 [0.86, 1.43]	**1.26 [1.06, 1.49]**
	Model 2	1.17 [0.96, 1.43]	1.07 [0.82, 1.40]	**1.23 [1.04, 1.47]**
	Model 3	1.17 [0.95, 1.43]	1.06 [0.81, 1.38]	**1.20 [1.01, 1.43]**

*Note:* Model 1: Adjusted for age, race, sex, study site, and BMI categories; Model 2: Model 1 + education, smoking, total fat mass, mid‐thigh muscle area, poor appetite, Healthy Eating Index, daily calorie intake, dietary protein and fat intake adjusting for energy intake, physical activity energy expenditure, prevalent/history of CVD, cancer, hypertension, diabetes, pulmonary disease, and number of medication use; Model 3: Model 2 + Cystatin C. Bold values indicate *p* < 0.05.

**TABLE A10:** Association between unintentional weight loss (UWL) metabolite scores and health outcomes in the Health, Aging and Body Composition study participants stratified by race and sex groups.

Strata	Metabolite score	Mortality	Mobility limitation
White men	UWL metabolite score	**1.43 [1.29, 1.59]**	**1.19 [1.05, 1.36]**
	UWL lipid score	**1.17 [1.07, 1.28]**	1.11 [0.99, 1.24]
	UWL organic acid score	**1.22 [1.10, 1.35]**	**1.18 [1.04, 1.34]**
	UWL sugar score	**1.28 [1.14, 1.43]**	1.10 [0.96, 1.26]
	UWL “other” score	**1.20 [1.09, 1.32]**	1.00 [0.89, 1.13]
White women	UWL metabolite score	**1.44 [1.28, 1.62]**	**1.24 [1.07, 1.44]**
	UWL lipid score	**1.19 [1.06, 1.32]**	1.12 [0.98, 1.27]
	UWL organic acid score	**1.27 [1.14, 1.42]**	**1.14 [1.00, 1.30]**
	UWL sugar score	**1.14 [1.00, 1.30]**	1.06 [0.91, 1.23]
	UWL “other” score	**1.21 [1.08, 1.35]**	1.10 [0.95, 1.26]
Black men	UWL metabolite score	**1.32 [1.16, 1.50]**	1.19 [1.00, 1.43]
	UWL lipid score	**1.19 [1.03, 1.37]**	1.12 [0.93, 1.35]
	UWL organic acid score	**1.19 [1.04, 1.36]**	1.23 [1.00, 1.52]
	UWL sugar score	**1.17 [1.01, 1.36]**	1.02 [0.83, 1.25]
	UWL “other” score	**1.20 [1.06, 1.36]**	1.08 [0.92, 1.27]
Black women	UWL metabolite score	**1.35 [1.16, 1.56]**	1.06 [0.87, 1.28]
	UWL lipid score	**1.08 [0.93, 1.25]**	1.04 [0.86, 1.26]
	UWL organic acid score	**1.23 [1.09, 1.39]**	1.02 [0.85, 1.23]
	UWL sugar score	**1.09 [0.95, 1.26]**	0.98 [0.80, 1.19]
	UWL “other” score	**1.20 [1.05, 1.37]**	1.05 [0.87, 1.25]

*Note:* Models adjusted for age, study site, BMI categories, smoking, total fat mass, mid‐thigh muscle area, poor appetite, Healthy Eating Index, daily calorie intake, dietary protein and fat intake adjusting for energy intake, physical activity energy expenditure, prevalent/history of CVD, cancer, hypertension, diabetes, pulmonary disease, and number of medication uses. Bold values indicate *p* < 0.05.

**TABLE A11:** Mortality predictive power for demographics, weight change groups, the unintentional weight loss (UWL) metabolite score, clinical biomarkers, and easily measured risk factors using C‐index among Health ABC participants.

Model	Predictors	C‐index (95% CI)	
		Overall	Hold‐out group
Model 0	Age, race, sex, study site	0.607 (0.591, 0.623)**, ***	0.623 (0.596, 0.650)**, ***
Model 1	Age, race, sex, study site, weight change groups	0.618 (0.602, 0.634)*, **, ***	0.628 (0.601, 0.655)**, ***
Model 2	Age, race, sex, study site, UWL metabolite score	0.645 (0.630, 0.661)*	0.650 (0.625, 0.676)*
Model 3	Age, race, sex, study site, clinical biomarkers	0.638 (0.623, 0.654)*, ***	0.654, (0.628, 0.679)*
Model 4	Age, race, sex, study site, traditional risk factors	0.653 (0.638, 0.668)*, **	0.662 (0.638, 0.687)*
Model 5	Age, race, sex, study site, traditional risk factors, UWL metabolite score	0.671 (0.657, 0.686)*, **, ***	0.676 (0.652, 0.700)*, **, ***

*Note:* Clinical biomarkers included total cholesterol, fasting glucose, CRP, IL‐6, cystatin C; Traditional risk factors included education, smoking, appetite, HEI, calorie intake, energy expenditure, CVD, cancer, hypertension, diabetes, pulmonary disease, number of medications.

Abbreviation: CI = confidence interval.

* *p* < 0.05 compared to Model 0.

** *p* < 0.05 compared to Model 3.

*** *p* < 0.05 compared to Model 4.

**TABLE A12:** Attenuation of unintentional weight loss‐mortality associations from cox models after further adjusting for unintentional weight loss associated metabolites, metabolite score, and other risk factors in the Health, Aging and Body Composition study participants (*N* = 2286).

Model	Metabolites	HR [95% CI]	% attenuation
Model 0	—	1.60 [1.38, 1.85]	0.0%
Model 1	Homovanillic acid	1.57 [1.36, 1.81]	4.3%
Model 1	CAR (6:0)^a^, ^b^	1.60 [1.38, 1.85]	0.5%
Model 1	CAR (4:0(OH))^b^	1.56 [1.35, 1.80]	5.8%
Model 1	Tetradecanedioic acid	1.58 [1.37, 1.83]	2.8%
Model 1	Lauric acid^a^	1.59 [1.38, 1.84]	0.9%
Model 1	Juniperic acid^a^	1.59 [1.37, 1.84]	1.6%
Model 1	Hexadecanedioic acid^b^	1.57 [1.36, 1.81]	4.1%
Model 1	3‐Methyladipic acid or pimelic acid	1.57 [1.36, 1.82]	3.9%
Model 1	Sebacic acid^a^	1.59 [1.38, 1.84]	1.0%
Model 1	Suberic acid	1.59 [1.38, 1.84]	1.2%
Model 1	PC (30:1)	1.60 [1.39, 1.85]	−0.4%
Model 1	LPC (22:5)^a^	1.59 [1.38, 1.84]	1.0%
Model 1	LPE (20:4)^a^	1.60 [1.39, 1.85]	−0.5%
Model 1	TG (46:1)	1.60 [1.38, 1.85]	0.1%
Model 1	TG (44:0)	1.60 [1.38, 1.85]	−0.1%
Model 1	TG (46:0)	1.60 [1.38, 1.85]	0.5%
Model 1	N4‐Acetylcytidine^b^	1.55 [1.34, 1.80]	6.2%
Model 1	Pseudouridine	1.55 [1.34, 1.79]	7.3%
Model 1	Ornithine^a^	1.60 [1.38, 1.85]	0.0%
Model 1	N‐Acetylglutamic acid	1.56 [1.35, 1.81]	4.7%
Model 1	N‐Formylmethionine^b^	1.53 [1.32, 1.77]	10.0%
Model 1	Malic acid^a^, ^b^	1.53 [1.32, 1.77]	9.4%
Model 1	N1‐Acetylspermidine^a^ ^,^ ^b^	1.58 [1.36, 1.82]	3.1%
Model 1	Fumaric acid or maleic acid	1.53 [1.32, 1.77]	9.2%
Model 1	3S‐Hydroxyhexanoic acid	1.58 [1.37, 1.83]	2.3%
Model 1	3‐Hydroxyoctanoic acid	1.59 [1.37, 1.83]	1.8%
Model 1	2‐Hydroxyglutaric acid	1.58 [1.37, 1.83]	2.2%
Model 1	cis‐Aconitic acid	1.56 [1.35, 1.80]	5.5%
Model 1	Myo‐inositol	1.58 [1.36, 1.83]	3.0%
Model 1	Adonitol or Arabitol	1.56 [1.35, 1.81]	5.1%
Model 1	Sucrose or Lactose or Trehalose^a^, ^b^	1.56 [1.35, 1.80]	5.6%
Model 1	Sorbitol^a^	1.57 [1.36, 1.81]	4.3%
Model 1	Threitol^a^, ^b^	1.56 [1.34, 1.80]	6.0%
Model 1	Hexose^a^, ^b^	1.58 [1.37, 1.83]	2.0%
Model 1	Glucuronic acid^b^	1.55 [1.34, 1.79]	7.3%
Model 1	Glucosan or 3‐Hydroxymethylglutaric acid^b^	1.56 [1.35, 1.81]	4.9%
Model 1	Theophylline	1.58 [1.37, 1.83]	2.0%
Model 1	Quinolinic acid^a^, ^b^	1.56 [1.35, 1.81]	4.9%
Model 1	Carboxyibuprofen^a^	1.59 [1.38, 1.84]	0.8%
Model 1	PC (P‐38:6)/PC (O‐38:7)^b^	1.55 [1.34, 1.79]	6.7%
Model 1	PC (40:6)	1.54 [1.33, 1.79]	7.6%
Model 1	PC (40:9)	1.54 [1.33, 1.78]	8.8%
Model 1	PC (38:6)	1.53 [1.32, 1.77]	9.3%
Model 1	PC (36:0)	1.58 [1.36, 1.82]	3.2%
Model 1	LPC (24:0)	1.57 [1.36, 1.82]	3.9%
Model 1	PE (38:2)^b^	1.58 [1.36, 1.82]	3.3%
Model 1	PE (P‐38:6)/PE (O‐38:7)^b^	1.54 [1.33, 1.78]	8.7%
Model 1	PS (34:0)	1.57 [1.36, 1.81]	4.4%
Model 1	SM (d18:1/22:1)	1.55 [1.34, 1.80]	6.4%
Model 1	SM (d18:1/24:0)^a^	1.56 [1.35, 1.80]	5.3%
Model 1	SM (d18:1/22:0)	1.56 [1.35, 1.81]	5.2%
Model 1	SM (d18:1/16:1)^a^	1.57 [1.36, 1.81]	4.1%
Model 1	SM (d18:1/20:0)^b^	1.56 [1.35, 1.80]	5.4%
Model 1	CE (22:6)	1.56 [1.35, 1.81]	5.1%
Model 1	CE (18:0)	1.58 [1.36, 1.82]	3.0%
Model 1	CE (20:5)	1.56 [1.35, 1.81]	4.7%
Model 1	TG (53:3)^a^, ^b^	1.60 [1.38, 1.85]	0.4%
Model 1	TG (60:12)	1.56 [1.35, 1.81]	4.9%
Model 1	TG (58:10)	1.56 [1.35, 1.81]	5.0%
Model 1	TG (58:9)	1.57 [1.36, 1.82]	3.7%
Model 1	TG (54:5)^a^	1.60 [1.38, 1.85]	0.3%
Model 1	TG (58:11)	1.56 [1.35, 1.80]	5.5%
Model 1	TG (54:6)^b^	1.59 [1.37, 1.83]	1.9%
Model 1	TG (56:8)^a^	1.55 [1.34, 1.79]	6.8%
Model 1	TG (54:9)^b^	1.56 [1.35, 1.81]	5.0%
Model 1	TG (56:7)	1.58 [1.37, 1.83]	2.8%
Model 1	TG (56:10)	1.56 [1.35, 1.80]	5.5%
Model 1	Uridine^a^, ^b^	1.50 [1.29, 1.73]	14.1%
Model 1	ADP^b^	1.61 [1.39, 1.86]	−0.9%
Model 1	ATP^a^	1.60 [1.39, 1.85]	−0.3%
Model 1	Tryptophan^a^, ^b^	1.57 [1.36, 1.82]	4.0%
Model 1	Homoarginine^a^, ^b^	1.56 [1.35, 1.81]	4.7%
Model 1	Glyceric acid^a^, ^b^	1.59 [1.38, 1.84]	0.8%
Model 1	1‐Methyl nicotinamide^a^, ^b^	1.59 [1.37, 1.83]	1.9%
Model 1	Niacinamide^a^, ^b^	1.58 [1.37, 1.83]	2.1%
Model 1	Uracil	1.50 [1.30, 1.74]	13.3%
Model 1	Serotonin	1.60 [1.38, 1.85]	0.1%
Model 1 (mean)	—		4.0%
Model 2	UWL metabolite score	1.33 [1.15, 1.54]	39.3%
Model 2_1	UWL lipid score	1.55 [1.34, 1.79]	7.1%
Model 2_2	UWL organic acid score	1.48 [1.28, 1.72]	16.3%
Model 2_3	UWL sugar score	1.53 [1.32, 1.76]	10.2%
Model 2_4	UWL “other” score	1.48 [1.28, 1.72]	16.4%
Model 3	Risk factors	1.52 [1.30, 1.77]	11.1%
Model 4	UWL metabolite score and risk factors	1.33 [1.14, 1.56]	38.7%
Model 5	Clinical biomarkers	1.49 [1.28, 1.73]	15.7%
Model 6	Metabolites selected by outcome‐based LASSO‐Cox	1.31 [1.12, 1.52]	43.3%

*Note:* Model 0: adjusted for age, race, sex, BMI categories, and study site; Model 1: Model 0 + individual metabolites; Model 3: Model 0 + risk factors including education, smoking, fat mass, muscle area, appetite, HEI, calorie intake, protein intake, fat intake, energy expenditure, CVD, cancer, hypertension, diabetes, pulmonary disease, number of medications; Model 4: Model 3 + risk factors; Model 5: Model 0 + Clinical biomarkers including total cholesterol, fasting glucose, CRP, IL‐6, cystatin C. Color shade indicate the magnitude of % attenuation.

^a^ Model 2: Model 0 + UWL metabolite score (^a^ indicates metabolites included in the score).

^b^ Model 6: Model 0 + LASSO‐Cox selected metabolites (^b^).

**TABLE A13:** Mobility limitation predictive power for demographics, weight change groups, the UWL metabolite score, clinical biomarkers, and traditional risk factors using C‐index among Health ABC participants.

Model	Predictors	C‐index (95% CI)	
		Overall	Hold‐out group
Model 0	Age, race, sex, study site	0.561 (0.542, 0.581)**, ***	0.570 (0.537, 0.630)**, ***
Model 1	Age, race, sex, study site, Weight change groups	0.563 (0.543, 0.583)**, ***	0.576 (0.543, 0.609)**, ***
Model 2	Age, race, sex, study site, UWL metabolite score	0.578 (0.559, 0.598)*, **, ***	0.581 (0.546, 0.616)**, ***
Model 3	Age, race, sex, study site, clinical biomarkers	0.600 (0.581, 0.619)*, ***	0.613 (0.582, 0.644)*
Model 4	Age, race, sex, study site, traditional risk factors	0.624 (0.605, 0.642)*, **	0.619 (0.586, 0.652)*
Model 5	Age, race, sex, study site, traditional risk factors, UWL metabolite score	0.631 (0.612, 0.649)*, **, ***	0.626 (0.593, 0.659)*

*Note:* Clinical biomarkers included total cholesterol, fasting glucose, CRP, IL‐6, cystatin C; Traditional risk factors included education, smoking, appetite, HEI, calorie intake, energy expenditure, CVD, cancer, hypertension, diabetes, pulmonary disease, number of medications.

Abbreviation: CI = confidence interval.

* *p* < 0.05 compared to Model 0.

** *p* < 0.05 compared to Model 3.

*** *p* < 0.05 compared to Model 4.

**TABLE A14:** Attenuation of unintentional weight loss‐mobility limitation associations from cox models after further adjusting for unintentional weight loss associated metabolites, metabolite score, and other risk factors in the health, aging and body composition study participants (*N* = 1578).

Model	Metabolites	HR [95% CI]	% attenuation
Model 0	—	1.25 [1.03, 1.51]	0.0%
Model 1	Homovanillic acid^b^	1.25 [1.03, 1.51]	0.1%
Model 1	CAR (6:0)^a^, ^b^	1.27 [1.05, 1.54]	−10.3%
Model 1	CAR (4:0(OH))^b^	1.23 [1.02, 1.49]	4.5%
Model 1	Tetradecanedioic acid	1.25 [1.03, 1.51]	0.4%
Model 1	Lauric acid^a^	1.25 [1.03, 1.51]	0.2%
Model 1	Juniperic acid^a^	1.25 [1.03, 1.51]	−0.2%
Model 1	Hexadecanedioic acid	1.24 [1.03, 1.50]	1.2%
Model 1	3‐Methyladipic acid or Pimelic acid	1.23 [1.02, 1.49]	4.6%
Model 1	Sebacic acid^a^	1.25 [1.03, 1.51]	0.0%
Model 1	Suberic acid	1.24 [1.03, 1.51]	0.6%
Model 1	PC (30:1)	1.25 [1.03, 1.51]	−0.3%
Model 1	LPC (22:5)^a^	1.24 [1.03, 1.50]	1.1%
Model 1	LPE (20:4)^a^	1.25 [1.03, 1.51]	−0.1%
Model 1	TG (46:1)	1.25 [1.03, 1.51]	−0.6%
Model 1	TG (44:0)	1.25 [1.03, 1.51]	−1.7%
Model 1	TG (46:0)	1.25 [1.03, 1.51]	0.1%
Model 1	N4‐Acetylcytidine^b^	1.24 [1.02, 1.50]	3.1%
Model 1	Pseudouridine	1.24 [1.03, 1.50]	1.1%
Model 1	Ornithine^a^	1.25 [1.03, 1.52]	−2.0%
Model 1	N‐Acetylglutamic acid^b^	1.24 [1.03, 1.51]	0.5%
Model 1	N‐Formylmethionine^b^	1.23 [1.01, 1.49]	7.2%
Model 1	Malic acid^a^	1.25 [1.03, 1.51]	0.4%
Model 1	N1‐Acetylspermidine^a^	1.24 [1.02, 1.50]	2.5%
Model 1	Fumaric acid or Maleic acid	1.25 [1.03, 1.51]	0.3%
Model 1	3S‐Hydroxyhexanoic acid	1.25 [1.03, 1.52]	−2.2%
Model 1	3‐Hydroxyoctanoic acid^b^	1.25 [1.03, 1.51]	−1.1%
Model 1	2‐Hydroxyglutaric acid	1.25 [1.03, 1.51]	−0.6%
Model 1	cis‐Aconitic acid	1.25 [1.04, 1.52]	−2.6%
Model 1	Myo‐inositol^b^	1.25 [1.03, 1.51]	−0.3%
Model 1	Adonitol or Arabitol	1.25 [1.03, 1.52]	−2.2%
Model 1	Sucrose or Lactose or Trehalose^a^	1.23 [1.01, 1.48]	7.3%
Model 1	Sorbitol^a^	1.24 [1.03, 1.50]	1.4%
Model 1	Threitol^a^	1.25 [1.03, 1.51]	−1.2%
Model 1	Hexose^a^, ^b^	1.25 [1.03, 1.51]	−1.5%
Model 1	Glucuronic acid^b^	1.23 [1.01, 1.48]	7.6%
Model 1	Glucosan or 3‐Hydroxymethylglutaric acid	1.26 [1.04, 1.52]	−3.5%
Model 1	Theophylline	1.25 [1.03, 1.51]	0.2%
Model 1	Quinolinic acid^a^, ^b^	1.23 [1.02, 1.49]	5.6%
Model 1	Carboxyibuprofen^a^	1.24 [1.03, 1.50]	1.7%
Model 1	PC (P‐38:6)/PC (O‐38:7)	1.23 [1.02, 1.49]	5.3%
Model 1	PC (40:6)	1.23 [1.01, 1.48]	7.4%
Model 1	PC (40:9)	1.22 [1.01, 1.48]	8.0%
Model 1	PC (38:6)	1.22 [1.01, 1.48]	7.9%
Model 1	PC (36:0)^b^	1.23 [1.02, 1.49]	4.5%
Model 1	LPC (24:0)^b^	1.22 [1.01, 1.48]	9.0%
Model 1	PE (38:2)	1.24 [1.03, 1.50]	2.1%
Model 1	PE (P‐38:6)/PE (O‐38:7)	1.23 [1.02, 1.49]	4.6%
Model 1	PS (34:0)	1.23 [1.02, 1.49]	5.1%
Model 1	SM (d18:1/22:1)	1.23 [1.02, 1.49]	6.2%
Model 1	SM (d18:1/24:0)^a^	1.22 [1.01, 1.48]	9.4%
Model 1	SM (d18:1/22:0)	1.23 [1.01, 1.49]	7.2%
Model 1	SM (d18:1/16:1)^a^	1.24 [1.02, 1.50]	2.8%
Model 1	SM (d18:1/20:0)^b^	1.23 [1.02, 1.49]	5.8%
Model 1	CE (22:6)	1.23 [1.02, 1.49]	6.2%
Model 1	CE (18:0)	1.24 [1.03, 1.50]	1.4%
Model 1	CE (20:5)	1.23 [1.01, 1.49]	7.0%
Model 1	TG (53:3)^a^, ^b^	1.25 [1.03, 1.51]	0.1%
Model 1	TG (60:12)	1.23 [1.02, 1.49]	6.4%
Model 1	TG (58:10)	1.23 [1.02, 1.49]	6.3%
Model 1	TG (58:9)^b^	1.23 [1.02, 1.49]	5.8%
Model 1	TG (54:5)^a^	1.25 [1.03, 1.51]	0.0%
Model 1	TG (58:11)^b^	1.23 [1.02, 1.49]	6.4%
Model 1	TG (54:6)	1.24 [1.03, 1.50]	1.4%
Model 1	TG (56:8)^a^	1.23 [1.01, 1.49]	7.0%
Model 1	TG (54:9)	1.23 [1.01, 1.49]	6.7%
Model 1	TG (56:7)	1.24 [1.02, 1.50]	4.1%
Model 1	TG (56:10)	1.23 [1.02, 1.49]	6.3%
Model 1	Uridine^a^, ^b^	1.21 [1.00, 1.47]	11.8%
Model 1	ADP	1.25 [1.03, 1.51]	−1.3%
Model 1	ATP^a^	1.25 [1.03, 1.51]	−0.5%
Model 1	Tryptophan^a^, ^b^	1.24 [1.03, 1.50]	1.8%
Model 1	Homoarginine^a^	1.24 [1.02, 1.50]	3.0%
Model 1	Glyceric acid^a^	1.24 [1.03, 1.51]	0.9%
Model 1	1‐Methyl nicotinamide^a^	1.25 [1.03, 1.51]	0.3%
Model 1	Niacinamide^a^	1.25 [1.03, 1.51]	−1.0%
Model 1	Uracil	1.22 [1.00, 1.47]	10.8%
Model 1	Serotonin^b^	1.25 [1.03, 1.52]	−2.0%
Model 1 (mean)	—		2.5%
Model 2	UWL metabolite score	1.17 [0.97, 1.42]	27.4%
Model 2_1	UWL lipid score	1.24 [1.02, 1.50]	3.6%
Model 2_2	UWL organic acid score	1.22 [1.01, 1.47]	10.4%
Model 2_3	UWL sugar score	1.23 [1.01, 1.48]	7.4%
Model 2_4	UWL “other” score	1.21 [1.00, 1.47]	12.3%
Model 3	Risk factors	1.17 [0.96, 1.43]	27.7%
Model 4	UWL metabolite score and risk factors	1.12 [0.91, 1.37]	50.2%
Model 5	Clinical biomarkers	1.19 [0.98, 1.45]	19.6%
Model 6	Metabolites selected by LASSO‐Cox	1.18 [0.97, 1.43]	26.7%

*Note:* Model 0: adjusted for age, race, sex, BMI categories, and study site; Model 1: Model 0 + individual metabolites; Model 3: Model 0 + risk factors including education, smoking, fat mass, muscle area, appetite, HEI, calorie intake, protein intake, fat intake, energy expenditure, CVD, cancer, hypertension, diabetes, pulmonary disease, number of medications; Model 4: Model 3 + risk factors; Model 5: Model 0 + Clinical biomarkers including total cholesterol, fasting glucose, CRP, IL‐6, cystatin C. Color shade indicate the magnitude of % attenuation.

^a^ Model 2: Model 0 + metabolite score (^a^ indicates metabolites included in the score).

^b^ Model 6: Model 0 + LASSO‐Cox selected metabolites (^b^).

**TABLE A15:** Association between unintentional weight loss (UWL) metabolite scores and health outcomes in the Health, Aging and Body Composition study participants stratified by weight change groups.

Strata	Metabolite score	Mortality	Mobility limitation
Unintentional weight loss	UWL metabolite score	**1.33 [1.16, 1.52]**	**1.51 [1.21, 1.90]**
	UWL lipid score	1.13 [0.99, 1.29]	1.18 [0.99, 1.42]
	UWL organic acid score	**1.25 [1.08, 1.44]**	**1.61 [1.28, 2.03]**
	UWL sugar score	**1.25 [1.07, 1.47]**	1.18 [0.94, 1.47]
	UWL “other” score	**1.15 [1.01, 1.31]**	1.07 [0.87, 1.30]
Intentional weight loss	UWL metabolite score	1.28 [0.98, 1.67]	1.24 [0.82, 1.87]
	UWL lipid score	0.88 [0.68, 1.14]	0.94 [0.66, 1.34]
	UWL organic acid score	**1.46 [1.16, 1.83]**	1.11 [0.83, 1.47]
	UWL sugar score	1.00 [0.78, 1.29]	1.29 [0.86, 1.93]
	UWL “other” score	1.12 [0.90, 1.39]	1.14 [0.82, 1.59]
Weight gain	UWL metabolite score	**1.29 [1.12, 1.49]**	1.19 [0.97, 1.46]
	UWL lipid score	1.06 [0.92, 1.22]	0.91 [0.76, 1.09]
	UWL organic acid score	**1.17 [1.03, 1.33]**	**1.21 [1.02, 1.45]**
	UWL sugar score	0.97 [0.84, 1.12]	1.15 [0.96, 1.39]
	UWL “other” score	**1.37 [1.18, 1.59]**	1.19 [0.97, 1.45]
Weight stable	UWL metabolite score	**1.41 [1.30, 1.54]**	**1.17 [1.06, 1.30]**
	UWL lipid score	**1.19 [1.10, 1.28]**	**1.13 [1.04, 1.24]**
	UWL organic acid score	**1.20 [1.11, 1.30]**	1.10 [1.00, 1.21]
	UWL sugar score	**1.25 [1.14, 1.37]**	1.03 [0.92, 1.15]
	UWL “other” score	**1.18 [1.09, 1.28]**	1.03 [0.94, 1.13]

*Note:* Models adjusted for age, study site, BMI categories, smoking, total fat mass, mid‐thigh muscle area, poor appetite, Healthy Eating Index, daily calorie intake, dietary protein and fat intake adjusting for energy intake, physical activity energy expenditure, prevalent/history of CVD, cancer, hypertension, diabetes, pulmonary disease, and number of medication use. Bold values indicate *p* < 0.05.
